# Transient knockdown and overexpression reveal a developmental role for the zebrafish *enosf1b *gene

**DOI:** 10.1186/2045-3701-1-32

**Published:** 2011-09-26

**Authors:** Steve Finckbeiner, Pin-Joe Ko, Blake Carrington, Raman Sood, Kenneth Gross, Bruce Dolnick, Janice Sufrin, Paul Liu

**Affiliations:** 1 Oncogenesis and Development Section, National Human Genome Research Institute, 49 Convent Drive, Bethesda MD, 20892, USA; 2 Program of Molecular Pharmacology and Cancer Therapeutics, Roswell Park Graduate Division, State University of New York at Buffalo, Elm and Carlton Streets, Buffalo NY, 14263, USA; 3 Zebrafish Core, National Human Genome Research Institute, 49 Convent Drive, Bethesda MD, 20892, USA; 4 Department of Molecular and Cellular Biology, Roswell Park Cancer Institute, Elm and Carlton Streets, Buffalo NY, 14263, USA; 5 Department of Pharmacology and Therapeutics, Roswell Park Cancer Institute, Elm and Carlton Streets, Buffalo NY, 14263, USA

## Abstract

**Background:**

Despite detailed *in vivo *knowledge of glycolytic enolases and many bacterial non-enolase members of the superfamily, little is known about the *in vivo *function of vertebrate non-enolase enolase superfamily members (ENOSF1s). Results of previous studies suggest involvement of the β splice form of ENOSF1 in breast and colon cancers. This study used the zebrafish (*Danio rerio*) as a vertebrate model of ENOSF1β function.

**Results:**

Whole mount in situ hybridization (WISH) showed that zebrafish ENOSF1β (*enosf1b*) is zygotic and expressed ubiquitously through the first 24 hours post fertilization (hpf). After 24 hpf, *enosf1b *expression is restricted to the notochord. Embryos injected with *enosf1b*-EGFP mRNA grew slower than EGFP mRNA-injected embryos but caught up to the EGFP-injected embryos by 48 hpf. Embryos injected with ATG or exon 10 *enosf1b *mRNA-targeting morpholinos had kinked notochords, shortened anterior-posterior axes, and circulatory edema. WISH for *ntl *or *pax2a *expression showed that embryos injected with either morpholino have deformed notochord and pronephros. TUNEL staining revealed increased apoptosis in the peri-notochord region.

**Conclusions:**

This study is the first report of ENOSF1 function in a vertebrate and shows that ENOSF1 is required for embryonic development. Increased apoptosis following *enosf1b *knockdown suggests a potential survival advantage for increased ENOSF1β expression in human cancers.

## Background

Sequence information and computational techniques can be used to group proteins into evolutionarily meaningful families and larger superfamilies. Dayhoff defined protein families as groups of proteins with high (>50%) sequence identity [1]. Members of protein superfamilies have lower sequence identities, but statistically significant pairwise alignment scores. Both protein families and protein superfamilies are thought to be monophyletic [1-4]. Since Dayhoff's original work, newer semi-automated classification schemes revealed thousands of protein superfamilies [5,6]. The enolase superfamily (ENOSF), named after the enolase of glycolysis, is used as a model of protein superfamily evolution [4,7]. Members of the enolase superfamily share a common ENOSF fold and catalyze a common half reaction: they all abstract protons adjacent to carboxyl groups from a wide array of substrates. Evolutionarily conserved acidic residues, located in loops at the end of two of the β sheets lining the C-terminal barrel of the ENOSF fold, coordinate an essential magnesium and are shared by all ENOSFs. Within the superfamily, different ENOSF families are distinguishable by the identity of the third magnesium ligand and by the different combinations of general acid/base catalytic residues at the ends of the remaining β sheets in the C-terminal barrel [4,7].

Two of the ENOSF families have representatives in eukaryotes. The ubiquitous glycolytic enolases reversibly dehydrate 2-phosphogylcerate to phosphoenolpyruvate [7,8]. Despite being named for mandelate racemase (MR), most members of the MR family with measured enzymatic activities and x-ray structures are acid sugar dehydratases. While many bacterial MR family members have known activities and even x-ray structures with bound ligands and inhibitors [9-20], little is known about what roles MR family members perform in eukaryotes. Some fungi have catalytically active MR family members that participate in a sugar metabolizing pathway that allows these fungi to use the sugar rhamnose as a sole carbon source [21,22]. The next best studied group of eukaryotic MR family members are splice isoforms of the human ENOSF1 protein. Human cancer cell lines have three known ENOSF1 splice forms (α, β, and γ). Immunoblotting with polyclonal and monoclonal antibodies shows that the β isoform (hsENOSF1β) is the major protein product of the human ENOSF1 locus [23,24]. In cell culture, hsENOSF1β expression appears to be regulated by environmental factors such as cell density, time in culture, and exposure to chemotherapy drugs that affect thymidylate synthase or folate pathway enzymes [23-27]. Using patient samples from a Taiwanese population, Kuo *et al *[28] showed that hsENOSF1β is expressed in breast tumor tissue and not surrounding tissue. The α or γ splice forms were not found in the patient samples [28]. The same group also reported a statistically significant decrease in five year survival of colon cancer patients with tumor hsENOSF1β expression compared to colon cancer patients without tumor hsENOSF1β expression [29]. Taken together, *in vitro *cell culture data [23-27] and human patient data [28,29] suggest a connection between hsENOSF1β expression and cancer.

In this study, we used the zebrafish (*Danio rerio*) as an *in vivo *model of ENOSF1β expression. Small adult size, ease of spawning, and rapidly developing glass-clear embryos make zebrafish attractive as a vertebrate model for the study of genes with unknown function. Zebrafish also have a sequenced genome, allowing for easy gene finding and straightforward comparison of zebrafish genes to human genes [30-33]. Building on a previously published bioinformatics study [23], homology searches of the zebrafish genome show that zebrafish have an hsENOSF1β homologue (*enosf1b*). We show that *enosf1b *is expressed ubiquitously early in development and is then restricted primarily to notochord after the first 24 hours of development. Phenotypes resulting from *enosf1b *knockdown by microinjection of *enosf1b*-targeted morpholino oligonucleotides provide evidence for *enosf1b*'s involvement in normal development: *enosf1b*-knockdown embryos have deformed notochords. Further characterization with apoptosis and cell cycle markers show that *enosf1b*-knockdown embryos have increased cell death in the tissues surrounding the notochord, while mitosis appears to be unaffected.

## Results

### Phylogenetic analysis reveals a complicated evolutionary history for vertebrate ENOSF1β genes

**Figure 1:**
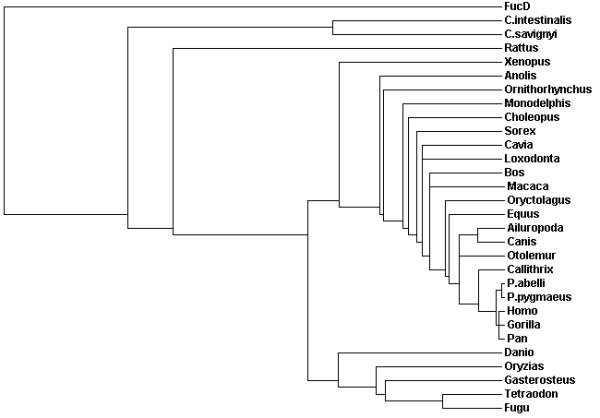
**Phylogenetic tree of full length cephalochordate and vertebrate hsENOSF1β homologues**. Protein sequences in Additional file 1 were aligned with MUSCLE and a guide tree was constructed automatically. *C. intestinalis *and *C. savignyi *are tunicates in the genus *Ciona*. *P. abelli *and *P. pygmaeus *are orang-utans in the genus *Pongo*.

Protein sequences used in the phylogenetic analysis can be found in Additional file 1. The complete MUSCLE alignment of all full length ENOSF1β homologues is found in Additional File 2. BLASTP searches of NCBI and Ensembl databases followed by MUSCLE alignment and phylogenetic tree construction reveal that the ENOSF1β gene was likely present in the last common ancestor of cephalochordates (*Ciona *sp, Figure 1) and vertebrates and then lost in different vertebrate classes. The anole (*Anolis*) has an ENOSF1β while no chicken (*Gallus*) or zebra finch (*Taeniopygia*) ENOSF1β homologues were found. With the exception of the guinea pig (*Cavia*), rodents as an order appear to have lost or are losing their ENOSF1β homologues. The kangaroo rat (*Dipodomys*) and 13-lined ground squirrel (*Spermophilus*) have predicted ENOSF1β genes that are missing exons found in other animals. Other sequences with missing exons (Additional file 3) were excluded from MUSCLE alignment and phylogenetic tree construction. While the rabbit (*Oryctolagus*), guinea pig (*Cavia*), rat (*Rattus*), and rhesus monkey (*Macaca*) genomes are predicted to contain a full length ENOSF1β homologue, their predicted sequences are so divergent that they branch away from their known evolutionary relatives (Figure 1). The mouse, pig, and sheep do not have identifiable ENOSF1β homologue sequences in their genomes.

### The zebrafish genome contains one full length homologue of hsENOSF1β

**Figure 2:**
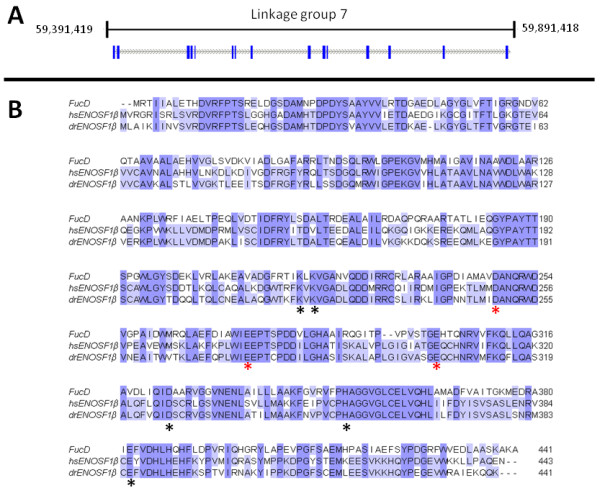
**The zebrafish homologue of hsENOSF1β**. A: Genomic context of *enosf1b*. Blue bars are exons, grey dashed lines are introns. B: Alignment of *enosf1b*, hsENOSF1β, and FucD. Residues marked with a black asterisk are involved in FucD proton abstraction. Conserved residues marked with a red asterisk stabilize the magnesium ion required for FucD's catalytic mechanism.

Using hsENOSF1β ([GenBank:NP_059982.2]) as the query sequence, BLASTP searches of the latest version of the zebrafish genome revealed a single full length homologue of human hsENOSF1β, [GenBank:NP_001070210.2**]**, which we have named *enosf1b*. Further bioinformatic searching of the zebrafish genome with Ensembl genome viewer revealed that *enosf1b *is located on linkage group 7, base pairs 59,391,419 through 59,891,418 (Figure 2A). The predicted zebrafish ENOSF1β (drENOSF1β) protein is 71% identical to hsENOSF1β at the protein level. BLASTP searching of the PDB [34] showed that the closest homologue of hsENOSF1β and drENOSF1β with a solved structure and known biochemical activity is fuconate dehydratase (FucD, [PDB:1YEY]) from the plant pathogenic bacteria *Xanthomonas campestris *[12]. Alignments of protein sequence with the bl2seq algorithm show that hsENOSF1β and drENOSF1β are 51% and 54% identical to FucD, respectively. MUSCLE alignment of hsENOSF1β, drENOSF1β, and FucD protein sequences (Figure 2B) show that both vertebrate ENOSF1β proteins share residues required for coordinating magnesium and proton abstraction in FucD [4,7,12].

### The zebrafish homologue of hsENOSF1 is expressed during development

**Figure 3:**
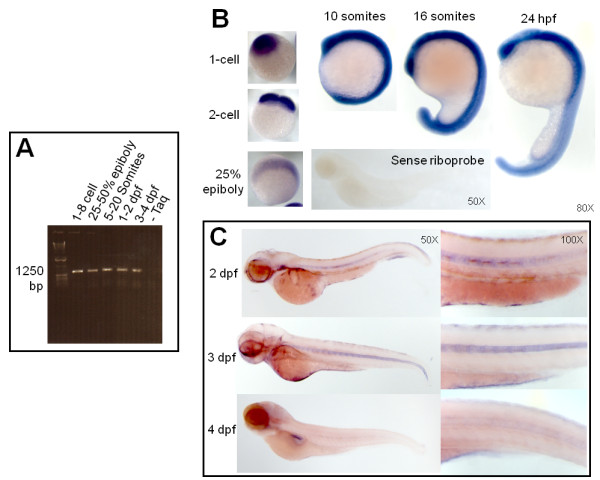
**Expression of *enosf1b *during development**. A: *enosf1b *expression measured by RT-PCR. Primers for full length *enosf1b *(Table 1) were used to test for the presence or absence of *enosf1b *in staged embryo lysate. Bp = base pairs. B: Whole mount in situ hybridization (WISH) with sense and antisense DIG-labelled riboprobe during early embryonic development. Negative control for WISH, DIG-labelled sense riboprobe, is free of WISH colored precipitate. C: WISH for *enosf1b *during later embryonic development. Except for panel C, original magnification for all photomicrographs is in the lower right hand corner of each picture.

Primers located at the first ATG in exon 1 and the 3' end of exon 15 (primers "*enosf1b *full length forward " and "*enosf1b *full length reverse", Table 1) were used to amplify *enosf1b *cDNA, with pooled RNA from multiple developmental stages. The result shows that *enosf1b *is expressed throughout early embryonic development (Figure 3A). Sequencing of the RT-PCR products cloned into pEGFP-N1 (p*-enosf1b*-N1-EGFP) confirmed the identity of *enosf1b *(data not shown).

**Table 1:** Oligonucleotide primers used in this study.

Primer name	Primer sequence
*Enosf1b *full length forward	ATGCTGGCGATCAAAATCATA

*Enosf1b *full length reverse	CTGCTGTTTCTCAATGGCTCT

*Enosf1b *XhoI Kozak forward	ATACTCGAGGACACCATGCTGGCGATCAAA

*Enosf1b *SacII reverse	CTCGAGCCGCGGATTTTTCTGCTGTTTCTC

Exon 10 flanking forward	GCCCTTGTGGAAGCTACTTG

Exon 10 flanking reverse	GTGGGGCTTTTGAAGTGTTC

WISH performed with *enosf1b *antisense probe on zygote through early cleavage stage embryos showed that *enosf1b *was zygotic and expressed throughout the early embryo (Figure 3B). Embryos undergoing early somitogenesis (10 somites) ubiquitously expressed *enosf1b*, while embryos in mid-somitogenesis (16 somites) had stronger expression in tissues such as the eye, somite borders, and notochord (Figure 3B). Ubiquitous *enosf1b *expression decreased while notochord *enosf1b *expression in 48 to 72 hpf embryos remained (Figure 3C). At 96 hpf, there was faint notochord *enosf1b *(see higher magnification panel in Figure 3C) expression and expression in the pancreas (Figure 3C).

### Overexpression of *enosf1b*-EGFP impairs embryonic development

**Figure 4:**
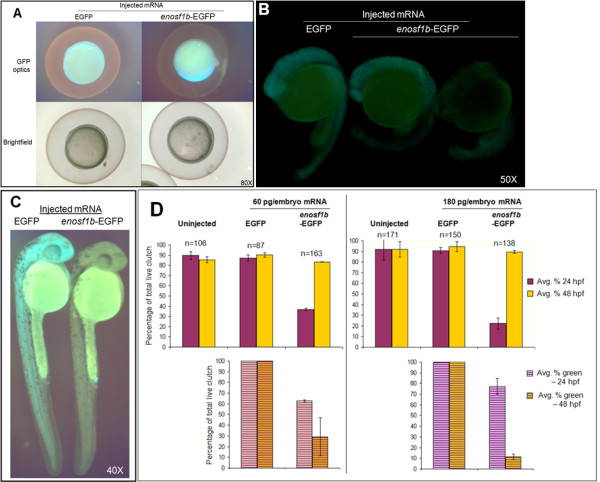
***In vivo *expression of *enosf1b*-EGFP compared to EGFP**. A-C: Lateral views of representative embryos injected with in vitro transcribed mRNA encoding *enosf1b*-EGFP or EGFP. Original magnification for all photomicrographs is in the lower right hand corner of each picture. D: Comparison of the effect of injecting equal doses of *enosf1b*-EGFP or EGFP mRNA on time embryos reach 24 hpf or 48 hpf developmental stage. Data is average of three independent experiments; "n" values are total of the three experiments. Error bars are standard deviation. Original magnification for all photomicrographs is in the lower right hand corner of each picture.

Following microinjection of EGFP mRNA, fluorescence was visible throughout the embryos by epiboly and through 48 hpf (Figure 4A-C). Embryos with microinjected *enosf1b*-EGFP mRNA demonstrated fluorescence only in earlier stages but not at 48 hpf (Figure 4C). When embryos were staged at 24 hpf, approximately 90% of uninjected and EGFP-injected embryos were the correct stage. Only 20-40% of the *enosf1b*-EGFP-injected embryos had reached the 24 hpf stage (Figure 4D, top graphs); suggesting that they were developmentally delayed. This delay was more severe when a higher dose of *enosf1b*-EGFP was injected (40% for 60 pg/embryo and 20% for 180 pg/embryo). When embryos of all three groups were staged at 48 hpf, however, the delay had disappeared. Interestingly, the proportionate recovery of correct stage by *enosf1b*-EGFP-injected embryos at 48 hpf was accompanied by the loss of *enosf1b*-EGFP fluorescence (Figure 4D, bottom graphs), which may suggest that overexpressing *enosf1b *is not compatible with normal embryonic growth.

### Two separate *enosf1b*-targeting morpholinos generate similar dose-dependent phenotypes

**Figure 5:**
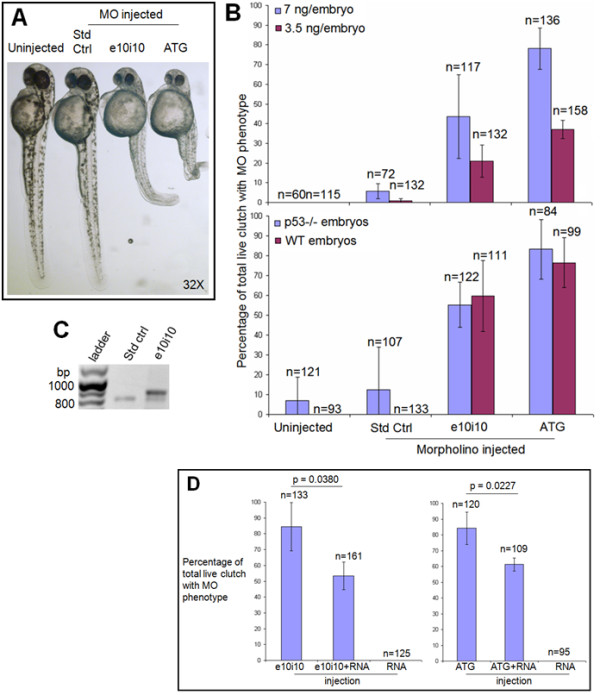
**Morpholino (MO) antisense oligonucleotide knockdown of *enosf1b *expression**. A: MO phenotypes. Photomicrograph of uninjected, standard control (Std Ctrl), e10i10, or ATG2 MO-injected embryos at 48 hpf. Original magnification is in the lower right hand corner. B (top panel): Effect of injecting three morpholinos at two different doses compared to uninjected embryos. B (bottom panel): Morpholino phenotypes are not dependent on p53 status. Data in B is average of three independent experiments; "n" values reported are total of the three experiments. Embryos were scored for MO phenotypes at 48 hpf. C: RT-PCR of uninjected, standard control, and e10i10 injected single embryos. Morpholinos were both injected at a final concentration of 7 ng/embryo. Single embryos were collected at 48 hpf and processed through RT-PCR as described in Methods. Bp = base pairs. D (left panel): Rescue of e10i10 phenotype by coinjection of e10i10 morpholino and *enosf1b*-EGFP mRNA. D (right panel): Titration of ATG2 phenotype by coinjecting ATG2 morpholino and *enosf1b*-EGFP mRNA. See Methods for MO and RNA doses. Data is average of three independent experiments; "n" values are total of the three experiments. Error bars are standard deviation. Embryos were scored at 48 hpf. Unpaired Student's t test was used to evaluate statistical significance of observed differences.

Embryos injected with *enosf1b *ATG MO at either 3.5 or 7 ng/embryo developed severe developmental defects when compared to uninjected or standard control-injected embryos. ATG MO-injected embryos had a shortened anterior-posterior axis, pericardial edema, and kinked notochords (Figure 5A). Embryos injected with *enosf1b *e10i10 MO at the same concentrations developed a similar phenotype, but of lesser severity (Figure 5A). When compared to clutches of embryos injected with 3.5 ng of either MO, clutches of embryos injected with 7 ng of either MO had a higher percentage of embryos with the respective MO phenotypes: the percentage of embryos with either phenotype for the respective MO is dose dependent (Figure 5B, top panel). Embryos with the phenotype of either MO died by 48 hpf.

### The e10i10 morpholino causes mis-splicing of *enosf1b*

RT-PCR was used to assay the presence or absence of altered *enosf1b *transcripts following injection of the e10i10 MO. Using primers that flank exon 10 (primer sequences in Table 1), PCR of cDNA from individual standard control MO or e10i10 MO-injected embryos (7ng/embryo, all at 48 hpf) revealed the presence of an additional PCR product ~100 bp longer than the 804 bp expected product (Figure 5C). Sequencing of the longer PCR product revealed insertion of intron 10 (Additional file 4). This confirmed the presence of mis-splicing induced by the e10i10 morpholino. As intron 10 contains an even number of base pairs (76 bp), its insertion alters the reading frame of the 3' end of *enosf1b *mRNA.

### Injecting morpholinos into p53 mutant embryos does not alter the ATG or e10i10 morpholino-induced phenotypes

The ATG, e10i10, and standard control MOs (all 7 ng/embryo) were injected into p53^-/- ^mutant embryos [35] and control wild-type embryos. As shown in Figure 5B (bottom panel), there is no statistically significant difference between the percentage of embryos with the ATG or e10i10 phenotype in injected p53 mutant embryos when compared to injected wild type embryos.

### The morpholino phenotypes are specific to knock down of *enosf1b*

Embryos were injected with *enosf1b*-EGFP mRNA alone (60 pg/embryo), either ATG or e10i10 MO alone (7 ng/embryo), or a combination of mRNA and one of the MOs (final concentrations of 60 pg/embryo *enosf1b*-EGFP mRNA and 7 ng/embryo MO). Figure 5D (left panel) shows that coinjecting *enosf1b*-EGFP mRNA with the e10i10 MO decreased the percentage of embryos with the e10i10 phenotype. Results of unpaired Student's t test show that this decrease is statistically significant (p value = 0.0380). In addition, coinjecting *enosf1b*-EGFP mRNA and the ATG MO also decreased the percentage of the embryos with the ATG phenotype (Figure 5D, right panel, p = 0.0227).

### Notochord and pronephros is deformed in both *enosf1b *morphants

**Figure 6:**
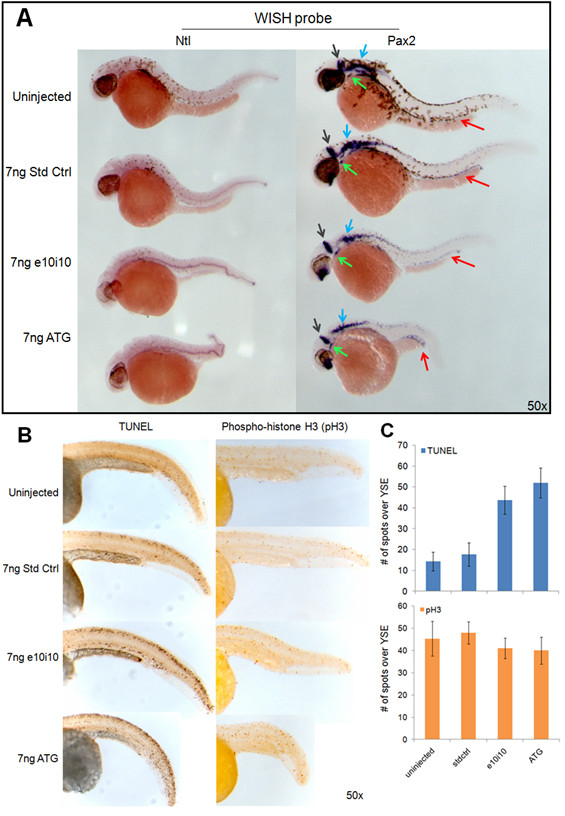
**Characterizing the *enosf1b *knockdown phenotype**. A: WISH for *no tail *and *pax2a *on 36 hpf morpholino-injected embryos. Red, grey, blue, and green arrows indicate *pax2a *staining of the pronephros, midbrain-hindbrain boundary, prospective cranial nerves, and thyroid primordium respectively. B (left panel): TUNEL staining on uninjected, std ctrl, e10i10, and ATG2 injected 48 hpf embryos. B (right panel): Mitotic index measured in uninjected, std ctrl, e10i10, and ATG2 injected embryos by antibody staining for phospho-histone H3. C: Morpholino-injected embryos have increased TUNEL staining but unchanged pH3 staining. Data is average of 3 tail counts per condition. Error bars are standard deviation. YSE = yolk sac extension. Original magnification for all photomicrographs is in the lower right hand corner of each picture.

The notochord phenotype was confirmed by WISH for the notochord-specific transcription factor *no tail *(*ntl*) (Figure 6A, left). While approximately the same diameter and overall length, the notochord in e10i10 and ATG MO-injected embryos is kinked multiple times. The expression of a gene with a wider distribution pattern, *pax2a*, was unaffected in the brain and thymus primordium, slightly reduced in the prospective cranial nerves and eye, but expanded in the pronephros in the ATG morpholino-injected embryos (Figure 6A, right).

### Knockdown of *enosf1b *increases apoptosis but does not change mitosis during zebrafish development

Results of TUNEL staining show that the area immediately surrounding the notochord had more apoptotic cells in ATG and e10i10 MO-injected embryos when compared to standard control injected and uninjected embryos (Figure 6B-C). On the other hand, antibody staining for a specific marker of mitotic cells, phosphorylated histone H3, did not change across the different experimental groups (Figure 6B-C).

## Discussion

A previously published phylogenetics study of the ENOSF1 gene [23] found ENOSF1 homologues in all the major phyla except plants. Expanding on these results, we used bioinformatics to find more animal homologues of the ENOSF1 splice form associated with increased cancer risk in human patients: hsENOSF1β [28,29]. The overall topology of the ENOSF1β tree (Figure 1) follows the generally accepted model for evolution within chordates [36-40]. Birds apparently lost their ENOSF1βs sometime after the last common ancestor of reptiles and birds (Figure 1). Within the mammalian clade, the branching pattern is dominated more by missing species and rodents that branch within other orders. The rat and guinea pig ENOSF1βs should cluster together with the rabbit ENOSF1β as outgroup. All three sequences should be a sister group to the primates ([36,41], but see: [42,43]). The missing mouse ENOSF1β, the diverged rat ENOSF1β, and "normal" ENOSF1β in the guinea pig support the hypothesis that rodents are losing their ENOSF1β genes. This is not as unusual as it may first seem. A recently published study details the loss of the motilin gene and its receptor during rodent evolution [44]. On a broader scale, the work of Hahn and colleagues measures this kind of loss (and gain) across mammalian lineages and entire gene families [45].

Of the vertebrates typically used as model systems with a clearly identifiable genomic ENOSF1β, the only diploid species suitable for small scale laboratory work are the zebrafish, *Danio rerio*, and the frog, *Xenopus tropicalis*. *X. tropicalis *has less of the developed molecular biology resources that make zebrafish such a widely used model vertebrate [46]. Homology searches of the zebrafish genome show that zebrafish have a hsENOSF1β homologue (*enosf1b*), the protein product of which (drENOSF1β) is predicted to be 71% identical to human hsENOSF1β. The predicted drENOSF1β has all the key features of the human cancer-associated gene as well as a bacterial gene with a known function: drENOSF1β and hsENOSF1β are both close homologues (slightly > 50% sequence identity) to the bacterial protein fuconate dehydratase (FucD) and share the conserved residues needed for that enzyme's catalytic activity (Figure 2B) [4,7,12].

Results of RT-PCR and WISH experiments also confirm that *enosf1b *is expressed during development (Figure 3A-C). The pattern of *enosf1b *loss during development suggests involvement of drENOSF1 in notochord function [47-49]. It should be noted that there is still faint notochord expression at 4 dpf (see higher magnification inset in 3C) that does not photograph well. While it appears that the notochord signal is disappearing, it is possible that the decreased *enosf1b *staining is due to lack of probe penetration. Embryos at 4 dpf are beginning to grow skin and are known for having weaker staining of more medial tissues and higher relative viscera staining [50].

Expression of *enosf1b *was manipulated during zebrafish development by injecting *enosf1b*-EGFP mRNA or antisense morpholino oligonucleotides into 1-2 cell stage embryos. Injection of *enosf1b*-EGFP mRNA into 1-2 cell embryos led to a relatively weak gain-of-function phenotype: developmental delay at 24 hpf (Figure 4A-C). While EGFP-injected embryos were essentially identical to uninjected embryos, significantly fewer *enosf1b*-EGFP-injected embryos had progressed to the same stage of development as the control embryos. Interestingly, the *enosf1b*-EGFP-injected embryos caught up to the EGFP-injected and uninjected embryos at 48 hpf, exactly when the *enosf1b*-EGFP signal disappeared (Figure 4D). The relatively mild gain-of-function phenotype can be explained by instability of the *enosf1b*-EGFP mRNA and/or the *enosf1b*-EGFP protein [51,52].

Morpholino antisense oligonucleotides were used to generate *enosf1b *loss-of-function phenotypes. Multiple lines of evidence show that the knockdown phenotypes observed are specific to *enosf1b *knockdown. First, two morpholinos that target different evolutionarily conserved regions of the *enosf1b *transcript led to similar phenotypes (Additional file 5 and Figure 5A). The second line of evidence supporting the specificity of the observed knockdown phenotypes is provided by RT-PCR for *enosf1b *following injection of the e10i10 morpholino. Single embryo RT-PCR shows that embryos with the e10i10 phenotype express a longer transcript (Figure 5C). Sequencing the longer product revealed that intron 10 is inserted into the mis-spliced e10i10 transcript (see Additional file 4). Insertion of intron 10 should cause frame shift of the remaining coding sequence. This effectively removes 4 of the 8 evolutionarily conserved catalytic residues (see Figure 2). The third line of evidence supporting the *enosf1b*-specificity of the morpholino phenotypes is provided by the results of injecting the morpholinos into p53 mutant embryos. Some morpholino phenotypes, particularly brain apoptosis, were shown to be caused by a p53-mediated antisense response [53]. The p53 mutant line expresses the full length p53 but has a mutation in the transactivation domain and cannot initiate p53-dependent transcription [35]. *Enosf1b*-targeting morpholino-injected p53 mutant embryos are indistinguishable from morpholino-injected wild type embryos (Figure 5B, bottom panel). The fourth line of evidence supporting the *enosf1b*-specificity of the morpholino-induced phenotypes is the results of the mRNA-morpholino coinjection, or rescue, experiments. Coinjecting *enosf1b *and either of the two morpholinos "rescues" or decreases the morpholino-induced phenotype (Figures 5D).

The most striking visibly observable characteristic of both morpholino-induced phenotypes is the kinked notochord. WISH shows that *enosf1b *is strongly expressed in notochord between late somitogenesis and 48 hpf. WISH for a notochord-specific gene, *no tail *[54], clearly highlights the severely deformed notochord in *enosf1b *morpholino knockdown embryos (Figure 6A). The salient feature of the knockdown phenotypes produced by different anti-*enosf1b*, a kinked notochord by 48 hpf, is precisely where and when *enosf1b *is highly expressed. WISH for *Pax2a *[55] was also performed. *Pax2a *is a transcription factor that is involved in development of the eye, brain, cranial neurons, thyroid primordium, and pronephros. The WISH data for pax2a suggests that *enosf1b *is involved in the normal development of some of those tissues.

Other assays performed on anti-*enosf1b *morpholino-injected embryos also reveal changes in the peri-notochord tissues. When compared to standard control injected or uninjected embryos, anti-*enosf1b *morpholino-injected embryos have increased TUNEL staining [56] in the peri-notochord tissues. The increased apoptosis is apparently not dependent on a stalled cell cycle. Whole mount antibody staining for an accepted proliferation marker, phospho-histone H3 (pH3) shows that there is no visible change in the cell cycle in *enosf1b*-knockdown embryos (Figure 6B). Experimental embryos had equivalent pH3 staining, indicating no change in achieving M-phase. The TUNEL and phospho-histone H3 (pH3) results for uninjected embryos shown in Figure 6B are similar to TUNEL results reported elsewhere [56,57]. Additional support for a cell cycle-independent increase in apoptosis is provided by the previously described p53-independence of the morpholino-induced phenotypes (Figure 5B, bottom panel). Increased apoptosis following *enosf1b *knockdown is especially interesting given the recent clinical studies implicating human ENOSF1β as a risk factor for two separate cancers [28,29]. Perhaps increased ENOSF1β confers a survival advantage on tumour cells by interfering with apoptosis [58-61].

The increased apoptosis is limited to the area around the notochord (see especially e10i10 panel in Figure 6B). There are two possible explanations for this change. First, *enosf1b *could be involved in production of a pro-survival signal or consumption of a pro-death signal by the notochord. When *enosf1b*-targeting morpholinos are injected, *enosf1b *decreases and the pro-survival signal disappears or the pro-death signal increases, leading to increased apoptosis. This idea fits with previous discovery of a soluble signal produced by hsENOSF1β-overexpressing drug resistant cancer cells [62] and the known signalling role of the notochord. In addition to providing structural support for the developing embryo, the notochord secretes soluble signalling proteins like hedgehog which is known to be involved in inducing formation of the ventral neural tube [49,63]. Notochord-produced hedgehog probably also plays a role in somite differentiation and inhibition of cardiac mesoderm fate in paraxial mesoderm. Removing the notochord, or interfering with hedgehog signalling, also interferes with pancreas development [48,49]. In addition to hedgehog, notochords of different species are known to secrete many soluble proteins and growth factors like BMPs, TGFβs, and FGFs [47,49]. A simpler but no less interesting possibility can explain the increased peri-notochord increase in apoptosis: that is where *enosf1b *expression is low enough to be affected by morpholino knockdown. Cells with decreased *enosf1b *cannot survive and die.

## Conclusions

Given the recent publications identifying hsENOSF1β expression as a risk factor in at least two human cancers [28,64], developing an *in vivo *animal model of ENOSF1β takes on added importance. The results of the WISH study presented here represent the first time a vertebrate ENOSF1β has been visualized in an intact model organism. The results of the mRNA and morpholino experiments are the first overexpression and knockdown phenotypes reported for a vertebrate ENOSF1β. One of our major findings, increased apoptosis following *in vivo *ENOSF1 knockdown, has implications for cancer therapy. Given the results of the clinical studies of [28,64] and *in vitro *cell culture data [23-27,62,65], it is not unreasonable to hypothesize that targeting ENOSF1 catalytic activity or possible interactions with other proteins may lead to increased apoptosis in tumours.

## Methods

### Phylogenetics and bioinformatics

BLASTP [66-68], used within NCBI map viewer [68] and Ensembl genome browser [69], was used to find the protein sequences of vertebrate homologues of hsENOSF1β in the latest genome version available. Ensembl genome browser was used to locate the chromosomal location of *enosf1b*, and to identify exon/intron boundaries [69]. Top scoring BLAST hits were used to search the Zebrafish Gene Collection (ZGC, [70]) full length clone library. The hsENOSF1β sequence, [Genbank:NP_059982.2], was used to search the Protein Data Bank [34] for homologues with known function. The program bl2seq was used to calculate percent identity [67,71]. MUSCLE (2 iterations, CLUSTALW output) was used to align nucleotide and protein sequences and to generate phylogenetic trees [72]. JalView was used to visualize alignments [73]. NEBCutter was used to plan subcloning experiments [74].

### Zebrafish husbandry

Zebrafish were maintained and used following approved NHGRI ACUC protocols. Adult wild type zebrafish (EK strain) and p53-/- mutant zebrafish [35] were maintained in a recirculating aquaculture system at 28.5°C and a 14:10 light:dark cycle per Westerfield [75]. Single adults were separated with plastic dividers in individual pair breeding boxes (Dura-Cross, LPS Inc. Rochester NY) the night before spawning. At first light, the divider was pulled and the adults were allowed to spawn naturally. Eggs were manually separated from the adults, rinsed free of faeces and debris, and raised at 28.5°C in embryo media (480 mg Instant Ocean salts, 16 mL 100% v/v methylene blue, 8 L system water, [75]) until they reached the desired stage.

### Whole mount In Situ Hybridization (WISH)

A ZGC cDNA clone corresponding to a truncated zebrafish homologue of human ENOSF1β (*enosf1b*) in the vector pME18S-FL (*p-enosf1b*) was purchased from a commercial supplier (OPEN Biosystems, Hunstville, AL). Following confirmatory sequencing in the NHGRI zebrafish core, MUSCLE alignment of this sequence to the identified full length zebrafish homologue of hsENOSF1β showed that the 3' end of this clone contained multiple gaps of non-existent homology and a polyA tail. As multiple regions with gaps and polyA tails are not conducive to successful WISH [50], a fragment of the clone with better homology to the 5' end of *enosf1b *was created. The XhoI-EcoRV fragment (532 bp) of p-*enosf1b *was subcloned into pBluescript SK(-) producing the plasmid used to make *enosf1b *riboprobe: pBS-*enosf1b*-5' (see Additional File 6 for MUSCLE alignments illustrating this process). Plasmids containing sequences for *no tail *[54] and *Pax2a *[55] were kind gifts of Dr. Milton English (NIH, National Eye Institute). Linearized plasmids were used to generate antisense or sense digoxigenin (DIG)-labelled riboprobes using T3 or T7 polymerase RNA labelling kits (Roche, Indianapolis, IN). Riboprobes were purified free of salts and enzymes using a Megaclear kit (Ambion, Austin TX). Embryos were staged according to Kimmel *et al *[76] and fixed in 4% paraformaldehyde in PBS overnight (4% PFA/PBS) at 4°C. Embryos younger than 24 hours post fertilization (hpf) were manually dechorionated with watchmaker forceps after fixation. All reagents for the WISH procedure were purchased from Sigma. WISH was performed as in Thisse and Thisse [50].

### Morpholino (MO) antisense oligonucleotide design

MOs were purchased as lyophilized powders from GeneTools (Philomath, Oregon). In addition to the FITC-tagged standard control MO (abbreviated here as "std ctrl,", see [77]), two MOs that target different evolutionarily conserved regions of the *enosf1b *transcript or pre-mRNA were also obtained (Table 2). The FITC-tagged MO designated "ATG" targets the conserved ATG in exon 2 of the processed *enosf1b *transcript. The FITC-tagged MO designated "e10i10" targets the exon10-intron10 splice junction (e10i10) in *enosf1b *pre-mRNA. Exon 10 was chosen for targeting by splice-blocking MO because it contains 3 conserved residues necessary for catalysis in other members of the MR family. Alignments of the protein products of the conserved regions targeted by the two morpholinos can be found in Additional file 5. Morpholino sequences (Table 2) were *in silico *tested for specificity by using the BLASTN program [67,71] to search for alternative targets in the zebrafish genome. Morpholinos were resuspended in RNase-free water and quantified using a NanoDrop ND-1000 (Thermo Scientific) following quantification instructions. Immediately before use, diluted MOs were heated at 65°C for 10 minutes. Diluted MOs were then stored at room temperature until injected.

**Table 2:** Morpholino antisense oligonucleotide (MO) sequences used in this study.

Name	MO sequence
Standard Control (std ctrl)	CCTCTTACCTCAGTTACAATTTATA

e10i10	GTTTACCTTAGAGATGGAAGCATGA

ATG2	CAGAATAATCTGGATCTGTGTGTGCAT

### RT-PCR

Twenty zebrafish embryos each at the following stages: 1-8 cell, 25-50% epiboly, 5-20 somites, 24-48 hpf, and 72-96 hpf were dechorionated in pronase when needed and anesthetized in Tricaine per Westerfield [75]. Embryos were rinsed 3 times in sterile phosphate buffered saline (PBS), drained of all residual PBS, snap frozen in liquid nitrogen, and stored at -80°C. To assay developmental expression of *enosf1b*, staged embryos were thawed on ice, homogenized in Trizol (Invitrogen, Carlsbad CA), and total RNA purified per Trizol package instructions. Total RNA (1 μg) was used to prepare cDNA using a SuperScript III kit (also Invitrogen). Full length protein coding sequence of the zebrafish homologue of hsENOSF1 was PCR-amplified ("Full length" primers in Table 1) using AmpliTaq DNA polymerase (Applied Biosystems, Foster City CA) and a step down PCR protocol: 94°C, 10 minutes; 40 cycles of 94°C/30 s, 62°C/30 s, 72°C/60 s (annealing temperature was decreased 0.5°C/cycle the first 7 cycles to a final annealing temperature of 58°C); 72°C for 60 s. To clone full length *enosf1b *for construction of an in vitro transcription vector, full length protein coding sequence of *enosf1b *was PCR amplified using a proof-reading polymerase (Platinum Pfx, Invitrogen) and primers designed to add a 5' XhoI site, a Kozak sequence, and a 3' SacII site to the PCR product (primer sequences in Table 1, PCR conditions same as above). To quantify mis-spliced *enosf1b *following e10i10 MO injection, single standard control injected or e10i10 MO-injected embryos were processed to total cDNA using a scaled down version of the Trizol protocol outlined above. Primers flanking *enosf1b *exon 10 (Table 1) were used in PCRs on the same samples [78]. Twenty percent of PCR reactions were run on precast 1% Tris-Borate-EDTA agarose gels containing ethidium bromide. Gels were photographed under UV light. Mis-spliced exon 10 PCR products were sequenced in the NHGRI zebrafish core.

### In vitro transcription of capped EGFP-polyA and *enosf1b*-EGFP-polyA mRNA

Following digestion with XhoI and SacII, *enosf1b *was ligated into pEGFP-N1 previously digested with XhoI and SacII, generating *enosf1b *fused in frame to the N-terminus of EGFP (*p-enosf1b-N1-EGFP*). *P*-*enosf1b*-N1-EGFP was sequenced in the NHGRI zebrafish core to verify that *enosf1b *was being amplified and for absence of PCR-induced mutations (sequencing data available upon request). The NheI/NotI fragment of p*-enosf1b*-N1-EGFP was subcloned downstream of the T7 promoter of pcDNA3.1(-) (*pcDNA3.1-enosf1b-EGFP-polyA*). The NheI/NotI fragment of pEGFP-N1 was also subcloned downstream of the T7 promoter of pcDNA3.1(-) (*pcDNA3.1-EGFP-polyA*).

Both pcDNA3.1-EGFP-polyA and pcDNA3.1-*enosf1b*-EGFP-polyA were linearized 3' of the polyadenylation signal sequence and phenol/chloroform extracted to remove restriction enzyme. The linearized plasmids were then ethanol precipitated (post-digest cleanup per [51]), resuspended in nuclease-free H_2_O, and used as templates for in vitro transcription (mMessage mMachine T7 Ultra *in vitro *transcription kit (Ambion, Austin TX)). Transcribed mRNA was phenol/chloroform extracted [78] and quantified on an ND-1000 Nanodrop spectrophotometer (Thermo Scientific). The mRNA was ethanol precipitated [78], resuspended in nuclease-free H_2_O, and stored in aliquots at -80°C until use.

### Embryo microinjection

Glass capillaries (World Precision Instruments, 1 mm inside diameter with filament) were pulled on a Kopf 730 vertical capillary puller. Using a sequencing gel loading pipette tip, capillaries were back-filled with *enosf1b*-GFP or GFP mRNA. Pulled capillaries were broken to a narrow tip with a razor blade under a stereomicroscope, calibrated in mineral oil [51], and used to inject 0.4 nL pulses of *in vitro *transcribed mRNA into the blastomeres of 1-2 cell embryos. The same procedure was used to prepare MO injection capillaries. For MO injection, the injection volume was adjusted to 1.4 nL and the injection target was the cytoplasmic streamers immediately ventral to the blastomeres of 1-2 cell embryos. Coinjections for rescue experiments used the injection volume for mRNA injection. Concentrations of stock mRNA and stock MOs were adjusted to achieve the final concentrations listed in Results above. Following microinjection, embryos were raised at 28.5°C and periodically photographed using a Leica MZ16F stereomicroscope with GFP epifluorescence optics.

### Scoring microinjection phenotypes and statistics

Fluorescence of EGFP and *enosf1b*-EGFP mRNA-injected embryos was scored at tailbud stage the night of the day of injection. GFP-negative embryos in injected clutches were discarded. GFP was assessed and stage was scored for surviving embryos at 24 and 48 hpf.

FITC-negative embryos in MO-injected clutches were discarded the night of injection. FITC-positive embryos were scored for MO-induced phenotypes daily until there were no surviving embryos (usually 48 hpf). "ATG phenotype" was defined as decreased anterior-posterior axis, severe notochord kinks and pericardial edema. "E10i10 phenotype" was defined as mild to severe notochord kinks and pericardial edema. Because the FITC-tagged MO masked the GFP signal, coinjection and MO-injected embryos were both treated as MO-injected the first day: fluorescence-negative embryos were discarded the night of the injection. Control mRNA-injected embryos were scored as described above. Percentage embryos in each clutch were scored for MO-induced phenotype using the criteria described above. All data values shown in the figures below are the average of three experiments with standard deviation bars. Total "n" values are the totals of the three independent experiments. When necessary, two-tailed Student's t test was used to calculate p values. All statistics and tests were calculated in Microsoft Excel.

### Whole mount TUNEL assay

The protocol used is modified from the package instructions of the Apoptag TUNEL kit (Chemicon, see also: [56,57]). Fixed 36 hpf embryos, in microcentrifuge tubes, were gradually dehydrated in five minute room temperature (RT) washes of 25%, 50%, 75%, ethanol/PBS. After two 100% ethanol washes, the embryos were stored overnight at -20°C. Embryos were then rehydrated to PBT (1X PBS, 0.1% Tween 20) by reversing the order of the washes above. Embryos were washed an additional 2 times in 100% PBT and then bleached in 3% peroxide in PBT for 10 minutes at RT. After bleaching, embryos were washed in PBT 3 times for 10 minutes each at RT. Embryos were then permeabilized by adding 5 uL of 2 mg/mL proteinase K to the final PBT wash (final volume 1 mL) and incubating for 10 minutes at RT. Proteinase K was aspirated and permeabilized embryos were washed twice in PBT (7 minutes at RT) and then post-fixed in 4% PFA/PBS for 15 minutes at RT. Trace amounts of PFA were removed by exhaustive PBT washes (five - five minute washes at RT). While washing in PBT, Apoptag equilibration and reaction buffers were thawed. Working enzyme solution was made (154 μL reaction buffer + 66 μL enzyme per tube assayed). The last of the 5 PBT washes was aspirated and replaced with equilibration buffer for 20 minutes at RT. Equilibration buffer was removed and replaced with working enzyme solution (50-100 μL of liquid per 20 embryos). Embryos in working enzyme solution were incubated on a heat block at 37°C for 60 minutes with 300 RPM agitation. While incubating in enzyme, Apoptag Stop/Wash concentrate was thawed and diluted to working Stop/Wash solution (1 mL concentrate + 34 mL ddH_2_O). Enzyme was removed and replaced with 1 mL of diluted Stop/Wash solution. Embryos were incubated in Stop/Wash solution at RT for 60 minutes with agitation. Stop/Wash solution was then washed off in 3 10 minute PBT washes with agitation. The last wash was aspirated and replaced with anti-DIG horseradish peroxidase conjugated monoclonal antibody (provided in the Apoptag kit) diluted 1:9,000 in blocking buffer (2% Roche blocking reagent, 10% lamb serum, and 1% DMSO in ddH_2_O). Embryos were incubated in anti-DIG antibody overnight at 4°C. Antibody was removed and embryos were washed 4 times in PBT for 10 minutes at RT. Washed embryos were stained in Tris-buffered DAB, (3,3'-Diaminobenzidine: SigmaFast, Sigma-Aldrich) for 5 minutes at RT in the dark. Immediately after staining, embryos were fixed in 4% PFA/PBS. Tails of TUNEL-stained embryos were photographed as described for WISH above. TUNEL-positive spots above the yolk sac extension (YSE) were counted for 3 embryos from each condition. Statistics were performed as above.

### Whole mount phosphohistone-H3 (pH3) antibody staining

PH3 staining protocol was developed from the protocol of [57]. Embryos (36 hpf) were fixed in 4% PFA/PBS as above. Following fixation, PFA/PBS was aspirated and embryos were incubated in ice cold acetone for seven minutes, followed by a brief rinse in ddH_2_O. Embryos were then washed in PBT (1X PBS, 0.1% Tween 20) two times for five minutes at RT. The last PBT wash was aspirated and replaced with blocking buffer (2% Roche blocking reagent, 10% lamb serum, and 1% DMSO in ddH_2_O). Embryos were blocked for 30 minutes at RT. While blocking, rabbit anti-pH3 polyclonal antibody (Santa Cruz Biotechnology) was diluted 1:200 in blocking buffer. After blocking, embryos were incubated in diluted anti-pH3 antibody overnight at 4°C. Antibody in blocking buffer was aspirated and embryos were washed in 4 15 minute PBT washes at RT. While washing, goat anti-rabbit HRP conjugated secondary antibody was diluted 1:1000 in blocking buffer. The last wash was aspirated and embryos were incubated in diluted anti-rabbit secondary antibody for two hours at RT with gentle agitation. Secondary antibody was aspirated and embryos were washed in 4 15 minute PBT washes at RT. Embryos were then stained in DAB, fixed in PFA, and photographed as described above for WISH. PH3-positive spots above the YSE were counted for 3 embryos from each condition. Statistics were performed as above.

## Competing interests

The authors declare that they have no competing interests.

## Authors' contributions

SF performed bioinformatics and phylogenetics studies. SF and PJK carried out WISH experiments. SF did embryo microinjection experiments, TUNEL and pH3 staining, and performed statistics. RS and SF designed morpholino sequences and oligonucleotide sequences used as primers. RS planned and guided sequencing experiments. BC performed sequencing experiments. SF, KG, BD, RS, JS, and PL conceived of the study and participated in the design of the study and helped to draft the manuscript. All authors approved the final manuscript.

## Supplementary Material

Additional file 1 **Protein sequences used in phylogenetic analysis**. NCBI or Ensembl accession numbers follow genus numbers. FucD is fuconate dehydratase of *Xanthomonas*. Click here for file

Additional file 2 **Alignment of protein sequences used in phylogenetic analysis**. Alignment done in MUSCLE. Click here for file

Additional file 3 **Protein sequences of predicted ENOSF1βs with missing exons and not used in phylogenetic analysis**. Ensembl accession numbers follow the genus name. Click here for file

Additional file 4 **E10i10-injected embryos express *enosf1b *transcript with inserted intron 10**. Sequence of mis-spliced product generated from e10i10-injected embryo cDNA and PCR with exon 10 flanking primers. Exons and introns are color-coded. Click here for file

Additional file 5 **Alignments of conserved regions targeted by morpholino antisense oligonucleotides used in this study**. Alignment done in MUSCLE. Click here for file

Additional file 6 **Creating the *enosf1b *WISH plasmid**. Sequences of *enosf1b *in plasmids p-*enosf1b *(Additional figure 1) and pBS-*enosf1b*-5' (Additional figure 2). See Methods for rationale. Click here for file
