# Supplementary material for: Transient knockdown and overexpression reveal a developmental role for the zebrafish enosf1b gene
Source: Cell Biosci. 2011 Sep 26;1:32. doi: 10.1186/2045-3701-1-32 (PMC3197473; doi:10.1186/2045-3701-1-32)
Supplement: Additional file 1 — Protein sequences used in phylogenetic analysis. NCBI or Ensembl accession numbers follow genus numbers. FucD is fuconate dehydratase of Xanthomonas. [file 2045-3701-1-32-S1.DOC]

**Additional file 1:** **Protein sequences used in phylogenetic analysis.**

NCBI or Ensembl accession numbers follow genus numbers. FucD is fuconate dehydratase of *Xanthomonas*.

>FucD PDB2HXT

MRTIIALETHDVRFPTSRELDGSDAMNPDPDYSAAYVVLRTDGAEDLAGYGLVFTIGRGNDVQTAAVAALAEHVVGLSVD

KVIADLGAFARRLTNDSQLRWLGPEKGVMHMAIGAVINAAWDLAARAANKPLWRFIAELTPEQLVDTIDFRYLSDALTRD

EALAILRDAQPQRAARTATLIEQGYPAYTTSPGWLGYSDEKLVRLAKEAVADGFRTIKLKVGANVQDDIRRCRLARAAIG

PDIAMAVDANQRWDVGPAIDWMRQLAEFDIAWIEEPTSPDDVLGHAAIRQGITPVPVSTGEHTQNRVVFKQLLQAGAVDL

IQIDAARVGGVNENLAILLLAAKFGVRVFPHAGGVGLCELVQHLAMADFVAITGKMEDRAIEFVDHLHQHFLDPVRIQHG

RYLAPEVPGFSAEMHPASIAEFSYPDGRFWVEDLAASKAKA

>Callithrix XP_002757153.1

MVRGRISRLSVRDVRFPTSLGGHGSDAMHTDPDYSAAYVVIETDAEDGLKGCGITFTLGKGTEVVVCAVN

ALAHHVLNKDLKDIVGDFRGFYRQLASDGQLRWIGPEKGVVHLATAAILNAVWDLWAKQEGKPVWKLLVD

MDPRTLVSCIDFRYITDVLTEEDALEILQKGQVGKKEREEQMLAQGYPAYTTSCAWLGYSDDTLKQLCTQ

ALKDGWTRFKVKVGADLQDDERRCQLIRDVIGPEKTLMMDANQRWDVPEAVEWMSKLAKFKPLWIEEPTS

PDDILGHATISKALVPLGIGVATGEQCHNRVIFKQLLQVKALQFLQIDSCRLGSINENLSVLLMAKKFEI

PVCPHAGGVGLCELVQHLIIFDYVSVSASLKNRMCEYVDHLHEHFKYPVVIQRASYMPPKDAGYSTEMKE

ESVKKHQYPDGEVWKKLLAAQEN

>Homo NP_059982.2

MVRGRISRLSVRDVRFPTSLGGHGADAMHTDPDYSAAYVVIETDAEDGIKGCGITFTLGKGTEVVVCAVN

ALAHHVLNKDLKDIVGDFRGFYRQLTSDGQLRWIGPEKGVVHLATAAVLNAVWDLWAKQEGKPVWKLLVD

MDPRMLVSCIDFRYITDVLTEEDALEILQKGQIGKKEREKQMLAQGYPAYTTSCAWLGYSDDTLKQLCAQ

ALKDGWTRFKVKVGADLQDDMRRCQIIRDMIGPEKTLMMDANQRWDVPEAVEWMSKLAKFKPLWIEEPTS

PDDILGHATISKALVPLGIGIATGEQCHNRVIFKQLLQAKALQFLQIDSCRLGSVNENLSVLLMAKKFEI

PVCPHAGGVGLCELVQHLIIFDYISVSASLENRVCEYVDHLHEHFKYPVMIQRASYMPPKDPGYSTEMKE

ESVKKHQYPDGEVWKKLLPAQEN

>Macaca XP_001088491.2

MVRGRISRLSVRDVRFPTSLGGHGSDAMHTDPDYSAAYVVIETDAEDGIKGCGITFTLGKGTEVDWSREG

RGAPGDSGCPKCGVGLVGQAGGKACLEVTCGHGETLVSCIDFRYITDVLTEEDALEILQKGQVGKKEREK

QMLAQGYPAYTTSCAWLGYSDDTLKQLCAQALKDGWTRFKVKVGADLQDDVRRCQIIRDMIGPEKTLMMD

ANQRWDVPEAVEWMSKLAKFKPLWIEEPTSPDDILGHAAISKALVPLGIGIATGEQCHNRVIFKQLLQAK

ALQFLQIDSCRLGSVNENLSVLLMAKKFEIPVCPHAGGVGLCELVQHLIIFDYISVSASLKNRMCEYVDH

LHEHFKYPVMIQRASYMPPKDPGYSTEMKEESVKKHQYPDGEVWKKLLAAQEN

>Pan XP_001149584.1

MVRGRISRLSVRDVRFPTSLGGHGSDAMHTDPDYSAAYVVIETDAEDGIKGCGITFTLGKGTEVVVCAVN

ALAHHVLNKDLKDIVGDFRGFYRQLTSDGQLRWIGPEKGVVHLATAAVLNAVWDLWAKQEGKPVWKLLVD

MDPRTLVSCIDFRYITDVLTEEDALEILQKGQVGKKEREKQMLAQGYPAYTTSCAWLGYSDDTLKQLCAQ

ALKDGWTRFKVKVGTDLQDDMRRCQIIRDMIGPEKTLMMDANQRWDVPEAVEWMSKLAKFKPLWIEEPTS

PDDILGHATISKALVPLGIGIATGEQCHNRVIFKQLLQAKALQFLQIDSCRLGSVNENLSVLLMAKKFEI

PVCPHAGGVGLCELVQHLIIFDYISVSASLENRVCEYVDHLHEHFKYPVMIQRASYMPPKDPGYSTEMKE

ESVKKHQYPDGEVWKKLLPAQEN

>P.abelli NP_001125659.1

MVRGRIFRLSVRDVRFPTSLGGHGSDAMHTDPDYSAAYVVIETDAEDGIKGCGITFTLGKGTEVVVCAVN

ALAHHVLNKDLKDIVGDFRGFYRQLTSDGQPRWIGPEKGVVHLATAAVLNAVWDLWAKQEGKPVWKLLVD

MDPRTLVSCIDFRYITDVLTEEDALEILQKGQVGKKEREKQMLAQGYPAYTTSCAWLGYSDDTLKQLCAQ

ALKDGWTRFKVKVGADLQDDVRRCQIIRDMIGLEKTLMMDANQRWDVPEAVEWMSKLAKFKPLWIEEPTS

PDDILGHATISKALVPLGIGIATGEQCHNRVIFKQLLQAKALQFLQIDSCRLGSVNENLSVLLMAKKFEI

PVCPHAGGVGLCELVQHLIIFDYISVSASLENRMCEYVDHLHEHFKYPVMIQRASYMPPKDPGYSTEMKE

ESVKKHQYPDGEVWKKLLAAQEN

>Ornithorhynchus XP_001508330.1

MATAYCILAVMHSIDPGLGSYRRGRLSLSVAELYFPRHKSHLANSLYRGMLFISVQHTDPDYSAAYVTLE

TDANDGLKGYGLTFTLGKGTEVVVCAVNALAHHVVNKDLAEIVSNFRGFYRQLTSDGQLRWIGPEKGVVH

LATAAILNAVWDLWAKQEGKPLWKLLVDMDPKQLLSCIDFRYITDALTEEEAYEILQKGLVGKKEREEQM

LKNGYPAYTTSCAWLGYSDQRLKQLCQKALEDGWTRFKVKVGADLQDDMRRCRLLRELIGPENILMLDAN

QRWDVQEAVEWVSKLAEFKPLWIEEPTSPDDILGHATISKALSPLGIGVATGEQCHNRVIFKQLLQAKAL

QYLQIDSCRLGSVNENLSVLLMAKKFQIPVCPHAGGVGLCELVQHLIIFDFISVSGSLENRMCEFVDHLH

EHFKYPVVIKKASYMPPQDPGYSSEMKEDSVKQHEFPEGEVWQNLMAAQKL

>Monodelphis XP_001363381.1

MVGGRITRLSVHDVRFPTSFEGHGSDAMHTDPDYSAAYVIIETDAKDGLKGYGITFTLGKGTEVVVCAIH

ALSHHVLNKNLGEIVRNFRGFYRQLTSDGQLRWIGPEKGVVHLATAAILNALWDLWAKQEGKPLWKLLVD

MDPKQLLSCIDFRYITDALTEEEAYEILQKGSIGKKEREKQMLEHGYPAYTTSCAWLGYSDQQLKQLCSE

ALKEGWTRFKVKVGADLEDDIRRCRIIRDMIGPEKTLMVDANQRWDVPEAVEWMLKLAEFKPLWIEEPTS

PDDILGHATISKALAPLGIGVATGEQCHNRVIFKQLLQAGALQFLQIDSCRLGSVNENLSVLLMAKKFQI

PVCPHAGGVGLCELVQHLIIFDYISISGSLENRMCEYVDHLHEHFKYPVVIKKASYMPPKDAGYSTEMKE

ESVKEHQFPEGKIWQKLMAAQKN

>Ailuropoda XP_002922664.1

MVRGRISRLSVRDVRFPTSLGGHGSDAMHTDPDYSAAYVVLETDVEDGLKGYGLTFTLGKGTEVVVCAVN

ALAHHVLNKDLRDIVGDFRGFYRQLTSDGQLRWIGPEKGVVHLATAAILNAVWDLWAKQEGKPLWKLLVD

MDPRTLLSCIDFRYITDVLTEEEAYEILQKGQVGKKEREGQMLMRGYPAYTTSCAWLGYSDDMLRQLCTE

ALKDGWTRFKVKVGADLQDDIRRCRLIRNMIGPEKTLMMDANQRWDVPEAIEWMSKLAEFKPLWIEEPTS

PDDILGHATISKALAPLGIGVATGEQCHNRVIFKQLLQAKALQFLQIDSCRLGSVNENLSVLLMAKKFEI

PVCPHAGGVGLCELVQHLIIFDFISVSASLRNRMCEYVNHLHEHFRYPVIIKKASYMPPKDAGYSTEMKE

ESIKKHQYPDGEVWKKLLAAQEN

>Bos NP_001040015.1

MVHGRVSRLSVHDVRFPTSLGGHGSDAMHTDPDYSAAYVVLETDAEDGLKGYGITFTLGRGTEVVVCAVN

ALAPHVLNKDLGEIVGDFRGFYRQLTSDGQLRWIGPEKGVVHLATAAVLNAVWDLWAKQEGKPLWKLLVD

MDPRTLVSCIDFRYITDVLTEEEACEILRQSQVGKKEREEQMLAHGYPAYTTSCAWLGYPDATLKQLCSE

ALKDGWTRFKVKVGADLQDDIRRCRLVRNMIGPEKTLMMDANQRWDVPEAVEWMTKLAEFKPLWIEEPTS

PDDILGHAAISKALAPLGIGVATGEQCHNRVIFKQLLQAKALKFLQIDSCRLGSVNENLSVLLMAKKFEI

PVCPHAGGVGLCELVQHLIIFDFISVSASLQDRMCEYVDHLHEHFKYPVLIREAAYMPPKDAGYSTEMKE

DSVKRHRYPDGEVWKKLLSAQGN

>Canis XP_848625.1

MVRGRICSLLVRDVRFPTSLGGHGSDAMHTDPDYSAAYVVLETDAEDGLKGYGITFTLGKGTEVVVCAVN

ALAHHVLNKDLSDIVGDFRGFYRQLTSDGQLRWIGPEKGVVHLATAAILNAVWDLWAKQEGKPLWKLLVD

MDPRTLLSCIDFRYITDVLTEEEAYEILQKGQVGKKEREGQMLMHGYPAYTTSCAWLGYSDDTLKQLCTE

ALKAGWTRFKVKVGADLQDDVRRCRLIRNMIGPEKTLMMDANQRWDVPEAVKWMSKLAEFKPLWIEEPTS

PDDILGHATISKALAPLGIGVATGEQCHNRVIFKQLLQAKALQFLQIDSCRLGSVNENLSVLLMAKKFEI

PVCPHAGGVGLCELVQHLIIFDFISISASLQNRMCEYVDHLHEHFRYPVIIKKASYMPPKDAGYSTEMKE

ESVKKHQYPDGEVWKKLLAAQEN

>Equus XP_001915737.1

MVRGRISGLSVRDVRFPTSLGGHGSDAMHTDPDYSAAYVVLETDAGDGLKGYGITFTLGKGTEVVVCAVN

ALAHHVLHKDLKDIVGDFRAFYRQLTSDGQLRWIGPEKGVVHLATAAVLNAVWDLWAKQEGKDPRTLLSC

IDFRYITDVLTEEEAFEILQKGQVGKKEREEQMLVQGYPAYTTSCAWLGYSDDTLKQVGLXPWFCRQVLY

SWWTLFKVKVGADLQDDIRRCRLIRNTIGPEKTLMMDANQRWDVPEAVEWMSKLAEFKPLWIEEPTSPDD

ILGHAAISKALVPLGIGVATGEQCHNRVIFKQLLQAKALQFLQIDSCRLGSVNENLSVLLMAKKFEIPVC

PHAGGVGLCELVQHLIIFDFISVSASLTNRMCEYVDHLHEHFKYPVVIEKASYMPPKDAGYSTEMKEDSV

KKHQYPDGEVWKKLLAARGN

>Danio NP_001070210.2

MLAIKIINVSVRDVRFPTSLEQHGSDAMHTDPDYSVAYVVLETDKAELKGYGLTFTVGRGTEIVVCAVKA

LSTLVVGKTLEEITSDFRGFYRLLSSDGQMRWIGPEKGVIHLATAAVLNAVWDLWARVERKPLWKLLVDM

DPAKLISCIDFRYLTDALTEQEALDILVKGKKDQKSREEQMLKEGYPAYTTSCAWLGYTDQQLTQLCNEA

LAQGWTKFKVKVGADLQDDIRRCSLIRKLIGPNNTLMIDANQRWDVNEAITWVTKLAEFQPLWIEEPTCP

DDILGHASISKALAPLGIGVASGEQCHNRVMFKQFLQASALQFVQIDSCRVGSVNENLATILMAAKFNVP

VCPHAGGVGLCELVQHLILFDYISVSASLSNRMCEFVDHLHEHFKSPTVIRNAKYIPPKDPGFSCEMLEE

SVKKHQYPEGEVWRAIEKQQK

>Xenopus tropicalis ENSXETP00000006867

MITGTITSLHVTDVRFPTSLDQHGSDAMHTDPDYSAAYIVIETDAADGLKGHGLTFTLGK

GTEIVVCAVRALSRHVIGKALGDIVNNFRDFYRQLTSDGQLRWIGPEKGAVQLATAAVLN

AVWDLWAKKEKKPLWKLLVDMDPHQLVSCIDFRYITDALTEEEALEILQNGKQGQRDREE

HMLRSGYPAYTTSCAWLGYSDEQLKKLCSDALKEGWTRFKVKVGADLKDDIRRCELIRGM

IGPDNIMMLDANQRWDVQEAISWVKDLAKYKPLWIEEPTSPDDILGHATISKALSPLNIG

VATGEQCHNRVMFKQFLQANALQYLQIDSCRLGSVNENLSVLLMSKKFNVPVCPHAGGVG

LCELVQHLILFDYISVSGSLDNRMCEYVDHLHEHFMYPVIISRAAYMPPKDPGYSTEMKD

ESVLQYQFPDGEIWQK

>Otolemur ENSOGAP00000003009

HTDPDYSAAYVIIETDAEDGLKGYGITFTLGKGTEVVVCAVNALAHHVLNKDLKDIVGDFRGFYRQLTSD

GQLRWIGPEKGVVHLATAAVLNAVWDLWAKQEGKPLWKLLVDMDPRTLVSCIDFRYITDVLTEEDAYEIL

QKGQVGKKEREGHMLAHGYPAYTTSCAWLGYSDDTLKQLCTEALKDGWTRFKVKVGADVQDDMRRCRLIR

DMIGPENTLMMDANQRWDVPEAVEWMSKLAEFKPLWIEEPTSPDDILGHAAISKCHNRVIFKQLLQANAL

QFLQIDSCRLGSVNENLSVLLMAKKFEIPVCPHAGGVGLCELVQHLIIFDYISVSASLENRMCEYVDHLH

EHFKYPVKIRQASYMPPMDAGYSTEMKEESVKKHQYPDGEVWKKLLATQEN

>Gorilla ENSGGOP00000019679

MVRGRISRLSVRDVRFPTSLGGHGSDAMHTDPDYSAAYVVIETDAEDGIKGCGITFTLGKGTEIVVCAVN

ALAHHVLNKDLKDIVGDFRGFYRQLTSDGQLRWIGPEKGVVHLATAAVLNAVWDLWAKQEGKPVWKLLVD

MDPRTLVSCIDFRYITDVLTEEDALEILQKGQVGKKEREKQMLAQGYPAYTTSCAWLGYSDDTLKQLCAQ

ALKDGWTRFKVKVGADLQDDMRRCQIIRDMIGPEKTLMMDANQRWDVPEAVEWMSKLAKFKPLWIEEPTS

PDDILGHATISRALVPLGIGIATGEQCHNRVIFKQLLQAKALQFLQIDSCRLGSVNENLSVLLMAKKFEI

PVCPHAGGVGLCELVQHLIIFDYISVSASLENRVCEYVDHLHEHFKYPVMIQQASYMPPKDPGYSTEMKE

ESVKKHQYPDGEVWKKLLPAQEN

>P.pygmaeus ENSPPYP00000010153

MVRGRISRLSVRDVRFPTSLGGHGSDAMHTDPDYSAAYVVIETDAEDGIKGCGITFTLGKGTEVVVCAVN

ALAHHVLNKDLKDIVGDFRGFYRQLTSDGQLRWIGPEKGVVHLATAAVLNAVWDLWAKQEGKPVWKLLVD

MDPRTLVSCIDFRYITDVLTEEDALEILQKGQVGKKEREKQMLAQGYPAYTTSCAWLGYSDDTLKQLCAQ

ALKDGWTRFKVKVGADLQDDVRRCQIIRDMIGLEKTLMMDANQRWDVPEAVEWMSKLAKFKPLWIEEPTS

PDDILGHATISKALVPLGIGIATGEQCHNRVIFKQLLQAKALQFLQIDSCRLGSVNENLSVLLMAKKFEI

PVCPHAGGVGLCELVQHLIIFDYISVSASLENRMCEYVDHLHEHFKYPVMIQRASYMPPKDPGYSTEMKE

ESVKKHQYPDGEVWKKLLAAQEN

>Oryctolagus ENSOCUP00000007495

MVRGRIIRLSVRDVRFPTSLGGHGSDAMHTDPDYSAAYVVIETDAGDGLAGCGITFTLGKGTEVVVCAVN

ALAHHVLHKDLRDIVGDFRGFYRQLTSDGQLRWIGPKKGVVHLATAAVLNAVWDLWAKQEGKPLWKLLVD

MDPRTLLSCIDFRYITDVLTEEDAYDILQQAQVGKKEREEQMLTHGYPAYTTSCAWLGYSDDTLKQRCAE

ALKDGWTRFKVKVGADLQDDMRRCRLIRDMIGPEKTLMMDANQRWDVPEAVEWMSKLAEFKPLWIEEPTS

PDDILGHATISKALVPLGIGVATGEQCHNRVIFKQLLQANALQFLQIDSCRLGSVNENLSVILMAKKFGI

PVCPHAGGVGLCELVQHLIIFDYIAVSASLKNRMCEYVDHLHEHFKYPVTIKQASYMPPKAAGYSTEMKE

ESVKKHQYPEGEVWKRLHAAHEN

>Cavia ENSCPOP00000014045

MVHGRVSRLLVHDVRFPTSLGGHGSDAMHTDPDYSVAYVVLETDAEDGLRGCGLTFTLGKGTEVVVCAVN

ALAHHVLHRDLKDIVGNFRGFYRQLTSDGQLRWIGPEKGAVHLATAAILNAVWDLWAKQEGKPLWKLLVD

MDPRTLLSCIDFRYITDALTEEDALEILQKGQVGKKEREEQILTHGYPAYTTSCAWLGYPDDLLKQLCAA

ALKDGWTRFKVKVGADLQDDMRRCRLIRDLIGPDRTLMMDANQRWDVPEAVAWMSKLAEFKPLWIEEPTS

PDDILGHAAIAKALAPLGIGVATGEQCHNRVVFKQLLQANALQFLQIDSCRLGSVNENLSVILMAKKFGI

PVCPHAGGVGLCELVQHLIIFDYISVSTSLQNRMCEYVDHLHEHFKYPVYIKQASYMPPQDAGYSTEMKE

DSVKKHQYPDGEVWRKLLAAQEN

>Rattus IPI00782687.2 KERGRICGLSVHNLRFSTSLGGHGSGATHTDPHCSAAYVIEINSEDGLKGSGIMRTLGKDPEIFVCVVNA

LHYMVHKGLRDVVGDFTGFCRQLTHDGQLRLVRKERCVERLARAAILNAVRDLWAKQKGKPLWKLRVDMD

PEMLLSCVDFLIDVLTKQGAYGELCNGQLGKKEREKQMLRHGYPAYTVSCAWLGYSDSTLKLLSQTCLKF

GKTHFYRKSRCSCQDDIQRCCLIRDMNGPEKTLGQDIMIWKLDESILWLMSILAEFKPLWIEKPTSQNDI

IGHATISEALAPLGIGIATGEQCHNRVMIKQLLHKALQLLQIDSCRLGSVKENLSVSLMPKKFGIHLPPC

WWSWTLNWLVQHLIIFDCQSLPAFKTGRMCEYVDHLHEHFKYPMVIKHASYLPPKDAGFSTKMK

>Choleopus ENSCHOP00000008480

SDAMVSAGHTDPDYSAAYVVIETDAGDGLEGCGLTFTLGKGTEVVVCAVNALAHHLLHKD

LGDIVSDFRSFYRQLTSDGQLRWIGPEKGVVHLATAAVLNAVWDLWAKQEGKPLWKLLVD

MDPKAVLSCIDFRYITDALTEEEAYEILQKGRVGNKEREEQMLTCGYPAYTLCTGALKDG

WTRFKVKVGADLQDDIRRCRLIRDMIGPHNTLMLDANQRWDVPEAVEWMLKLAEFKPLWI

EEPTSPDDILGHAAIAKCHNRVMFKQLLQAKALQFLQIDSCRLGSVNENLSVLLMAKKFG

IPVCPHAGGVGLCELVQHLILFDYISVSACLQNRMCEYVDHLHEHFKYPVTIKKASYMPP

KDAGYSTEMKEESVNEYQYPDGEVWKKLLAAQEN

>Sorex ENSSARP00000011195

HTDPDYSAAYVTLETDAADGLRGYGFTFTLGKGTEVVVCAVNALAHHVLNKDLKDIVRDF

RGFYRELTSDGQLRWIGPEKGVVHLATAAVLNAVWDLWAKQEGKLLVDMDPRTLLSCIDF

RYITDVLTEEEAYEILREGEAAKKEREKQMLAQGYPAYTTSCAWLGYSDDMLKQLCTKAL

KDGWTRFKVKVGGDIQDDIRRCRFIRNMIGPDKTLMLDANQRWDVPEAVEWMAKLAEFKP

LWIEEPTSPDDILGHAAISKCHNRVMFKQLLQAKALQFLQIDSCRLGSVNENLSVLLMAK

KFQIPVCPHAGGVGLCELVQHLIIFDFISISASLENRMCEYVDHLHEHFKYPVRIQKAAY

MPPKDPGYSTEMKEESIKQHQYPDGDVWKKLLAAQGN

>Loxodonta ENSLAFP00000019598

MVRGRICGLSARDVRFPTSLGGHGSDAMHTDPDYSAAYVILETDAGDGLKGYGITFTLGK

GTEVVICAVNALAHHVLNRDFGDIVGDFRGFYRQLTSDGQLRWIGPEKGVVHLATAAILN

ALWDLWAKQEGKPLWKLLVDMDPKTLLSCIDFRYISDALTEEEAYEILKKGRVGHREREA

QLLAHGYPAYTTSCAWLGYSDDTLKQLCSEALKEGWTRFKVKVGADLQDDIRRCRLIRDM

IGPEKTLMMDANQRWDVHEAVEWMSQLAEFKPLWIEEPTSPDDILGHAAISKALAPLGIG

VATGEQCHNRVIFKQLLQANALQFLQIDSCRLGSINENVSVLLMAQKFGIPVCPHAGGVG

LCELVQHLIIFDYIAVSASLENRMCEYVDHLHEHFKYPVIIKKASYMPPEDPGYSTEMKE

ESVKKHQFPDGEVWTKLLAGQEN

>Anolis ENSACAP00000008434

MARGRITALTVSDVRFPTSLDHHGSDAMHPDPDYSAAYVVIQTDASDGLKGYGLTFTLGK

GTEVVVCAINALSAHVVNRDLDEIISDFRGFYRQLTSDGQLRWIGPEKGAVHLATAAILN

AVWDLWAKQEGKPLWKLLVDMDPKQLLSCIDFRYITDALTEEEAYTILQKGLVGKKEREE

QMLKYGYPAYTTSCAWLGYPDQQLKQLCTEALKDGWTRFKVKVGADLQDDIRRCRLVREM

IGPENILMLDANQRWEVEEAIEWVTKLAEFKPLWIEEPTSPDDVLGHATISKALALLGIG

VATGEQCHNRVVFKQLLQAQALSYVQIDSCRLGSVNENLSVLLMAKKFQIPVCPHAGGVG

LCELVQHLIIFDYISISGSLENRMCEYVDHLHEHFKYPVVIKNASYMPPQAPGYSSEMKE

DSVRKYQFPQGEIWQKLLSDPQT

>Gasterosteus ENSGACP00000005457

MSHKITALTVRDVRFPTSVEQHGSDAMHTDPDYSAAYVVIHTACGLKGFGFTFTLGKGTQ

IVVCAVEAVATLVVGKSLQEIVSDFRGFYRLLTSDGQMRWLGPEKGVIQLAVAAVLNAVW

DLWARAEGKPLWKLLVDMDPRQLASCIDFRYITDALTEEEAVDMIVKAQEGKQQREDQML

KEGYPAYTTSCAWLGYPDQLLRQLCTDALKSGWTRFKVKVGGDLEDDVRRCRLIRQMIGP

ENTLMIDANQRWGVAEAISWVSRLAEFKPLWIEEPTSPDDILGHAAISKALAPLGIGVAT

GEQCHSRVMFKQFLQASALQFVQIDGCRVGSVNENLAVLLMAHKFQVPVCPHAGGVGLCE

LVQHLILFDYISVSGSLSNRMCEYVDHLHEHFTSPVVIRDAHYMPPKDPGFSCEMLESSV

QRHQYPEGEVWKLNISK

>Oryzias ENSORLP00000013925

MSPKIVRVSVRDVRFPTSLEQHGSDAMHTDPDYSAAYVVLETDGGLRGFGLTFTLGKGTE

IVVCAVQAMTGLVVGKSLEEIVRDFRGFYRLLTSDGQMRWLGPEKGVIHLATAALLNAVW

DLWARMEGKPLWKLLVDMSPERLVSCIDFRYITDVLTEEEALELLVKAQEGKRQREEEML

REGYPAYTTSCAWLGYFDQQLKQLCTDALKGGWTKFKVKVGADLDDDVRRCRLIRQMIGP

DNTLMIDANQRWDVSEAISWVSNLAEVKPLWIEEPTSPDDILGHAAISKALAPLGIGVAT

GEQCHNRVMFKQFLQAGALQFVQIDSCRLGSVNENLAVLLMAHKFRVPVCPHAGGVGLCE

LVQHLILFDYICVSASLSNRMCEYVDHLHEHFVCPVVIHNARYMPPKIPGYSCEMLESSV

KKHQYPDGDAWKLYPKK

>Tetraodon ENSTNIP00000020250

KMLPRIVKLAVTDVRFPTSLEQHGSDAMHTDPDYSAAYVVIDTDCGLKGFGLTFTLGKGT

EIVVCAVEALARLVVGKSLQEIVSDFGGFYRLLTSESQLRWLGPEKGVIHLASAAVLNAV

WDLWARAEGKPLWKLLVDMDPKQIVSCIDFRYITDALTEEEALDILLKAREGRKQREDQM

LREGYPAYTTSCAWLGYSDELLTQLCADALGNGWTKFKVKVGADLEDDRRRCRLIREMIG

PSNTLMMDANQRWDVAEAIRWVSSLADFKPLWIEEPTCPDDILGHAAISKALAPLGIGVA

SGEQCHNRVMFKQFLQASALFVQIDSCRLGSVNENLAVLLMAHKFQVPVCPHAGGVGLCE

LVQHLILFDYICVSGSLANRMCEYVDHLHEHFASPVVIRNGHYIAPEDIGYSCEMLESSV

QSHRYPEGDVWKANTSKPEVKMDTF

>Fugu ENSTRUP00000006084

MLHKIVKLTVMDVRFPTSSEQHGSDAMHTDPDYSAAYVVIETECGLKGFGLTFTLGKGTE

IVVCAVEALAKLVVGMSWQEIVSDFRGFYRLLTSESQLRWLGPEKGVIHLASAAVLNAVW

DLWARAEGKPLWKLLVDMDPKQIVSCIDFRYITDALTEEEALDILLKAREGRQQREDQML

REGYPAYTTSCAWLGYSDEQLTQLCTDALQKGWTKFKVKVGADLEDDRRRCRLLRKIIGQ

SNTLMIDANQRWDVAEAIRWVSSLAEFNPLWIEEPTCPDDILGHAAISKALAPLGIGVAS

GEQCQNRVMFKQFLQASALQFVQIDSCRLGSINENLAVLLMAHKFQVPVCPHAGGVGLCE

LVQHLSLFDYICVSGSLTNRMCEYVDHLHEHFASPVVIRNGHYMPPKDLGYSCEMLASSV

QAHRYPEGDVWKKTFLVVSSGN

>C.intestinalis ENSCINP00000026411

MEVTGKITKIDVCDVRFPTSLEHHGSDAMHGEVDYSAAYVTMATDADDVIGCGLTFSLGR

GNDILVKAIEVIRGHVIGRQLSDIYSNFGKFCREITQEGQLRWLGPEKGVIHMASAAIFN

ALWDLWGKKCGKPVWKLLAEMSPHEVVSLVDFSYITDVLTKDEALKLLTRNKGTQKEREL

ILMEKGFPAYTTSTAWLGYSDETLVKKCREALVEGWTKFKMKVGSNVDDDKRRAKLIRDE

IGYNCDLMMDANQKWDVKEAIEWMKQLVEFRPLWIEEPTCPDDVIGHATIAKALSPHNVG

VATGEQCQNRVVFKQLLQVDGLKFLQIDSCRVGSINENIAILLMAAKFNVPVCPHAGGVG

LCELVQHIIMFDYLCVSATNEQRMCEYVDHLHEHFIEPVVIQNTCYMPPKKPGYSSEMKK

DSVENYKFPDGKIWKDLIEGGKFVQ

>C.savignyi ENSCSAVP00000000726

ISGKITEVKVRDIRFPTSLEHHGSDAMHGEVDYSAAYVTVETDSNDDIIGCGITFSLGRG

NDILVKAIEAIQGLVIGRELSNIYSNFGKFWREITQEGQLRWLGPEKGVVHMAAAGMFNA

LWDLWGKKAGKPLWKLLADMSPLEVVNLVDFSYITDAITKQEAMDILTRNKTSQKEREDQ

LLKRGFPAYTTSTAWLGYSDETLVKKCQEALAQGWTKFKMKVGSDIDDDVRRANIIRDQI

GYDRDLMMDANQKWDVNEAIEWMKPLVKFRPLWIEEPTSPDDVIGHATIAKALKEHKVGV

ATGEQCQNRVIFKQLMQTNAVSFVQIDSCRVGSINENIAILLMAAKFNLPVCPHAGGVGL

CELVQHIIMFDYLCVSATTDGRVCEYVDHLHEHFVEPVRIKDASYMSPQKPGYSSEMKAE

SLDNYEFPNGTIWSNLIKEGKFKM
