# Supplementary material for: Transient knockdown and overexpression reveal a developmental role for the zebrafish enosf1b gene
Source: Cell Biosci. 2011 Sep 26;1:32. doi: 10.1186/2045-3701-1-32 (PMC3197473; doi:10.1186/2045-3701-1-32)
Supplement: Additional file 2 — Alignment of protein sequences used in phylogenetic analysis. Alignment done in MUSCLE. [file 2045-3701-1-32-S2.DOC]

**Additional file 2**: **Alignment of protein sequences used in phylogenetic analysis.**

Alignment done in MUSCLE.

FucD ----------------------MRTIIALETHDVRFPTSRELDGSD-----AM----NPD

C.intestinalis -------------------MEVTGKITKIDVCDVRFPTSLEHHGSD-----AM----HGE

C.savignyi ---------------------ISGKITEVKVRDIRFPTSLEHHGSD-----AM----HGE

Rattus ------------------------------------------------------------

Xenopus --------------------MITGTITSLHVTDVRFPTSLDQHGSD-----AM----HTD

Anolis --------------------MARGRITALTVSDVRFPTSLDHHGSD-----AM----HPD

Ornithorhynchus MATAYCILAVMHSIDPGLGSYRRGRLS-LSVAELYFPRHKSHLANSLYRGMLFISVQHTD

Monodelphis --------------------MVGGRITRLSVHDVRFPTSFEGHGSD-----AM----HTD

Choleopus --------------------------------------------SD-----AMVSAGHTD

Sorex ---------------------------------------------------------HTD

Cavia --------------------MVHGRVSRLLVHDVRFPTSLGGHGSD-----AM----HTD

Loxodonta --------------------MVRGRICGLSARDVRFPTSLGGHGSD-----AM----HTD

Bos --------------------MVHGRVSRLSVHDVRFPTSLGGHGSD-----AM----HTD

Macaca --------------------MVRGRISRLSVRDVRFPTSLGGHGSD-----AM----HTD

Oryctolagus --------------------MVRGRIIRLSVRDVRFPTSLGGHGSD-----AM----HTD

Equus --------------------MVRGRISGLSVRDVRFPTSLGGHGSD-----AM----HTD

Ailuropoda --------------------MVRGRISRLSVRDVRFPTSLGGHGSD-----AM----HTD

Canis --------------------MVRGRICSLLVRDVRFPTSLGGHGSD-----AM----HTD

Otolemur ---------------------------------------------------------HTD

Callithrix --------------------MVRGRISRLSVRDVRFPTSLGGHGSD-----AM----HTD

P.abelli --------------------MVRGRIFRLSVRDVRFPTSLGGHGSD-----AM----HTD

P.pygmaeus --------------------MVRGRISRLSVRDVRFPTSLGGHGSD-----AM----HTD

Homo --------------------MVRGRISRLSVRDVRFPTSLGGHGAD-----AM----HTD

Gorilla --------------------MVRGRISRLSVRDVRFPTSLGGHGSD-----AM----HTD

Pan --------------------MVRGRISRLSVRDVRFPTSLGGHGSD-----AM----HTD

Danio --------------------MLAIKIINVSVRDVRFPTSLEQHGSD-----AM----HTD

Oryzias --------------------M-SPKIVRVSVRDVRFPTSLEQHGSD-----AM----HTD

Gasterosteus --------------------MSH-KITALTVRDVRFPTSVEQHGSD-----AM----HTD

Tetraodon --------------------KMLPRIVKLAVTDVRFPTSLEQHGSD-----AM----HTD

Fugu --------------------MLH-KIVKLTVMDVRFPTSSEQHGSD-----AM----HTD

FucD PDYSAAYVVLRTDGAEDLAGYGLVFTIGRGNDVQTAAVAALAEHVVGLSVDKVIADLGAF

C.intestinalis VDYSAAYVTMATDADD-VIGCGLTFSLGRGNDILVKAIEVIRGHVIGRQLSDIYSNFGKF

C.savignyi VDYSAAYVTVETDSNDDIIGCGITFSLGRGNDILVKAIEAIQGLVIGRELSNIYSNFGKF

Rattus -----------------------------------------LHYMVHKGLRDVVGDFTGF

Xenopus PDYSAAYIVIETDAADGLKGHGLTFTLGKGTEIVVCAVRALSRHVIGKALGDIVNNFRDF

Anolis PDYSAAYVVIQTDASDGLKGYGLTFTLGKGTEVVVCAINALSAHVVNRDLDEIISDFRGF

Ornithorhynchus PDYSAAYVTLETDANDGLKGYGLTFTLGKGTEVVVCAVNALAHHVVNKDLAEIVSNFRGF

Monodelphis PDYSAAYVIIETDAKDGLKGYGITFTLGKGTEVVVCAIHALSHHVLNKNLGEIVRNFRGF

Choleopus PDYSAAYVVIETDAGDGLEGCGLTFTLGKGTEVVVCAVNALAHHLLHKDLGDIVSDFRSF

Sorex PDYSAAYVTLETDAADGLRGYGFTFTLGKGTEVVVCAVNALAHHVLNKDLKDIVRDFRGF

Cavia PDYSVAYVVLETDAEDGLRGCGLTFTLGKGTEVVVCAVNALAHHVLHRDLKDIVGNFRGF

Loxodonta PDYSAAYVILETDAGDGLKGYGITFTLGKGTEVVICAVNALAHHVLNRDFGDIVGDFRGF

Bos PDYSAAYVVLETDAEDGLKGYGITFTLGRGTEVVVCAVNALAPHVLNKDLGEIVGDFRGF

Macaca PDYSAAYVVIETDAEDGIKGCGITFTLGKGTEVD-----------WSREGRGAPGDSGC-

Oryctolagus PDYSAAYVVIETDAGDGLAGCGITFTLGKGTEVVVCAVNALAHHVLHKDLRDIVGDFRGF

Equus PDYSAAYVVLETDAGDGLKGYGITFTLGKGTEVVVCAVNALAHHVLHKDLKDIVGDFRAF

Ailuropoda PDYSAAYVVLETDVEDGLKGYGLTFTLGKGTEVVVCAVNALAHHVLNKDLRDIVGDFRGF

Canis PDYSAAYVVLETDAEDGLKGYGITFTLGKGTEVVVCAVNALAHHVLNKDLSDIVGDFRGF

Otolemur PDYSAAYVIIETDAEDGLKGYGITFTLGKGTEVVVCAVNALAHHVLNKDLKDIVGDFRGF

Callithrix PDYSAAYVVIETDAEDGLKGCGITFTLGKGTEVVVCAVNALAHHVLNKDLKDIVGDFRGF

P.abelli PDYSAAYVVIETDAEDGIKGCGITFTLGKGTEVVVCAVNALAHHVLNKDLKDIVGDFRGF

P.pygmaeus PDYSAAYVVIETDAEDGIKGCGITFTLGKGTEVVVCAVNALAHHVLNKDLKDIVGDFRGF

Homo PDYSAAYVVIETDAEDGIKGCGITFTLGKGTEVVVCAVNALAHHVLNKDLKDIVGDFRGF

Gorilla PDYSAAYVVIETDAEDGIKGCGITFTLGKGTEIVVCAVNALAHHVLNKDLKDIVGDFRGF

Pan PDYSAAYVVIETDAEDGIKGCGITFTLGKGTEVVVCAVNALAHHVLNKDLKDIVGDFRGF

Danio PDYSVAYVVLETDKAE-LKGYGLTFTVGRGTEIVVCAVKALSTLVVGKTLEEITSDFRGF

Oryzias PDYSAAYVVLETDG--GLRGFGLTFTLGKGTEIVVCAVQAMTGLVVGKSLEEIVRDFRGF

Gasterosteus PDYSAAYVVIHTAC--GLKGFGFTFTLGKGTQIVVCAVEAVATLVVGKSLQEIVSDFRGF

Tetraodon PDYSAAYVVIDTDC--GLKGFGLTFTLGKGTEIVVCAVEALARLVVGKSLQEIVSDFGGF

Fugu PDYSAAYVVIETEC--GLKGFGLTFTLGKGTEIVVCAVEALAKLVVGMSWQEIVSDFRGF

:

FucD ARRLTNDSQLRWLGPEKGVMHMAIGAVINAAWDLAARAANKPLWRFIAELTPEQLVDTID

C.intestinalis CREITQEGQLRWLGPEKGVIHMASAAIFNALWDLWGKKCGKPVWKLLAEMSPHEVVSLVD

C.savignyi WREITQEGQLRWLGPEKGVVHMAAAGMFNALWDLWGKKAGKPLWKLLADMSPLEVVNLVD

Rattus CRQLTHDGQLRLVRKERCVERLARAAILNAVRDLWAKQKGKPLWKLRVDMDPEMLLSCVD

Xenopus YRQLTSDGQLRWIGPEKGAVQLATAAVLNAVWDLWAKKEKKPLWKLLVDMDPHQLVSCID

Anolis YRQLTSDGQLRWIGPEKGAVHLATAAILNAVWDLWAKQEGKPLWKLLVDMDPKQLLSCID

Ornithorhynchus YRQLTSDGQLRWIGPEKGVVHLATAAILNAVWDLWAKQEGKPLWKLLVDMDPKQLLSCID

Monodelphis YRQLTSDGQLRWIGPEKGVVHLATAAILNALWDLWAKQEGKPLWKLLVDMDPKQLLSCID

Choleopus YRQLTSDGQLRWIGPEKGVVHLATAAVLNAVWDLWAKQEGKPLWKLLVDMDPKAVLSCID

Sorex YRELTSDGQLRWIGPEKGVVHLATAAVLNAVWDLWAKQEGK----LLVDMDPRTLLSCID

Cavia YRQLTSDGQLRWIGPEKGAVHLATAAILNAVWDLWAKQEGKPLWKLLVDMDPRTLLSCID

Loxodonta YRQLTSDGQLRWIGPEKGVVHLATAAILNALWDLWAKQEGKPLWKLLVDMDPKTLLSCID

Bos YRQLTSDGQLRWIGPEKGVVHLATAAVLNAVWDLWAKQEGKPLWKLLVDMDPRTLVSCID

Macaca --------------PKCGVGLVGQAG-------------GKACLEVTCG-HGETLVSCID

Oryctolagus YRQLTSDGQLRWIGPKKGVVHLATAAVLNAVWDLWAKQEGKPLWKLLVDMDPRTLLSCID

Equus YRQLTSDGQLRWIGPEKGVVHLATAAVLNAVWDLWAKQEGK---------DPRTLLSCID

Ailuropoda YRQLTSDGQLRWIGPEKGVVHLATAAILNAVWDLWAKQEGKPLWKLLVDMDPRTLLSCID

Canis YRQLTSDGQLRWIGPEKGVVHLATAAILNAVWDLWAKQEGKPLWKLLVDMDPRTLLSCID

Otolemur YRQLTSDGQLRWIGPEKGVVHLATAAVLNAVWDLWAKQEGKPLWKLLVDMDPRTLVSCID

Callithrix YRQLASDGQLRWIGPEKGVVHLATAAILNAVWDLWAKQEGKPVWKLLVDMDPRTLVSCID

P.abelli YRQLTSDGQPRWIGPEKGVVHLATAAVLNAVWDLWAKQEGKPVWKLLVDMDPRTLVSCID

P.pygmaeus YRQLTSDGQLRWIGPEKGVVHLATAAVLNAVWDLWAKQEGKPVWKLLVDMDPRTLVSCID

Homo YRQLTSDGQLRWIGPEKGVVHLATAAVLNAVWDLWAKQEGKPVWKLLVDMDPRMLVSCID

Gorilla YRQLTSDGQLRWIGPEKGVVHLATAAVLNAVWDLWAKQEGKPVWKLLVDMDPRTLVSCID

Pan YRQLTSDGQLRWIGPEKGVVHLATAAVLNAVWDLWAKQEGKPVWKLLVDMDPRTLVSCID

Danio YRLLSSDGQMRWIGPEKGVIHLATAAVLNAVWDLWARVERKPLWKLLVDMDPAKLISCID

Oryzias YRLLTSDGQMRWLGPEKGVIHLATAALLNAVWDLWARMEGKPLWKLLVDMSPERLVSCID

Gasterosteus YRLLTSDGQMRWLGPEKGVIQLAVAAVLNAVWDLWARAEGKPLWKLLVDMDPRQLASCID

Tetraodon YRLLTSESQLRWLGPEKGVIHLASAAVLNAVWDLWARAEGKPLWKLLVDMDPKQIVSCID

Fugu YRLLTSESQLRWLGPEKGVIHLASAAVLNAVWDLWARAEGKPLWKLLVDMDPKQIVSCID

: . :. .. * : . :*

FucD FRYLSDALTRDEALAILRDAQPQRAARTATLIEQGYPAYTTSPGWLGYSDEKLVR-----

C.intestinalis FSYITDVLTKDEALKLLTRNKGTQKERELILMEKGFPAYTTSTAWLGYSDETLVK-----

C.savignyi FSYITDAITKQEAMDILTRNKTSQKEREDQLLKRGFPAYTTSTAWLGYSDETLVK-----

Rattus F--LIDVLTKQGAYGELCNGQLGKKEREKQMLRHGYPAYTVSCAWLGYSDSTLKL-----

Xenopus FRYITDALTEEEALEILQNGKQGQRDREEHMLRSGYPAYTTSCAWLGYSDEQLKK-----

Anolis FRYITDALTEEEAYTILQKGLVGKKEREEQMLKYGYPAYTTSCAWLGYPDQQLKQ-----

Ornithorhynchus FRYITDALTEEEAYEILQKGLVGKKEREEQMLKNGYPAYTTSCAWLGYSDQRLKQ-----

Monodelphis FRYITDALTEEEAYEILQKGSIGKKEREKQMLEHGYPAYTTSCAWLGYSDQQLKQ-----

Choleopus FRYITDALTEEEAYEILQKGRVGNKEREEQMLTCGYPAYT--------------------

Sorex FRYITDVLTEEEAYEILREGEAAKKEREKQMLAQGYPAYTTSCAWLGYSDDMLKQ-----

Cavia FRYITDALTEEDALEILQKGQVGKKEREEQILTHGYPAYTTSCAWLGYPDDLLKQ-----

Loxodonta FRYISDALTEEEAYEILKKGRVGHREREAQLLAHGYPAYTTSCAWLGYSDDTLKQ-----

Bos FRYITDVLTEEEACEILRQSQVGKKEREEQMLAHGYPAYTTSCAWLGYPDATLKQ-----

Macaca FRYITDVLTEEDALEILQKGQVGKKEREKQMLAQGYPAYTTSCAWLGYSDDTLKQ-----

Oryctolagus FRYITDVLTEEDAYDILQQAQVGKKEREEQMLTHGYPAYTTSCAWLGYSDDTLKQ-----

Equus FRYITDVLTEEEAFEILQKGQVGKKEREEQMLVQGYPAYTTSCAWLGYSDDTLKQVGLXP

Ailuropoda FRYITDVLTEEEAYEILQKGQVGKKEREGQMLMRGYPAYTTSCAWLGYSDDMLRQ-----

Canis FRYITDVLTEEEAYEILQKGQVGKKEREGQMLMHGYPAYTTSCAWLGYSDDTLKQ-----

Otolemur FRYITDVLTEEDAYEILQKGQVGKKEREGHMLAHGYPAYTTSCAWLGYSDDTLKQ-----

Callithrix FRYITDVLTEEDALEILQKGQVGKKEREEQMLAQGYPAYTTSCAWLGYSDDTLKQ-----

P.abelli FRYITDVLTEEDALEILQKGQVGKKEREKQMLAQGYPAYTTSCAWLGYSDDTLKQ-----

P.pygmaeus FRYITDVLTEEDALEILQKGQVGKKEREKQMLAQGYPAYTTSCAWLGYSDDTLKQ-----

Homo FRYITDVLTEEDALEILQKGQIGKKEREKQMLAQGYPAYTTSCAWLGYSDDTLKQ-----

Gorilla FRYITDVLTEEDALEILQKGQVGKKEREKQMLAQGYPAYTTSCAWLGYSDDTLKQ-----

Pan FRYITDVLTEEDALEILQKGQVGKKEREKQMLAQGYPAYTTSCAWLGYSDDTLKQ-----

Danio FRYLTDALTEQEALDILVKGKKDQKSREEQMLKEGYPAYTTSCAWLGYTDQQLTQ-----

Oryzias FRYITDVLTEEEALELLVKAQEGKRQREEEMLREGYPAYTTSCAWLGYFDQQLKQ-----

Gasterosteus FRYITDALTEEEAVDMIVKAQEGKQQREDQMLKEGYPAYTTSCAWLGYPDQLLRQ-----

Tetraodon FRYITDALTEEEALDILLKAREGRKQREDQMLREGYPAYTTSCAWLGYSDELLTQ-----

Fugu FRYITDALTEEEALDILLKAREGRQQREDQMLREGYPAYTTSCAWLGYSDEQLTQ-----

* : *.:* : * : . * :: *:****

FucD -LAKEAVADGFRTIKLKVGANVQDDIRRCRLARAAIGPDIAMAVDANQRWDVGPAIDW-M

C.intestinalis -KCREALVEGWTKFKMKVGSNVDDDKRRAKLIRDEIGYNCDLMMDANQKWDVKEAIEW-M

C.savignyi -KCQEALAQGWTKFKMKVGSDIDDDVRRANIIRDQIGYDRDLMMDANQKWDVNEAIEW-M

Rattus -LSQTCLKFGKTHFYRKSRCSCQDDIQRCCLIRDMNGPEKTLGQDI-MIWKLDESILWLM

Xenopus -LCSDALKEGWTRFKVKVGADLKDDIRRCELIRGMIGPDNIMMLDANQRWDVQEAISW-V

Anolis -LCTEALKDGWTRFKVKVGADLQDDIRRCRLVREMIGPENILMLDANQRWEVEEAIEW-V

Ornithorhynchus -LCQKALEDGWTRFKVKVGADLQDDMRRCRLLRELIGPENILMLDANQRWDVQEAVEW-V

Monodelphis -LCSEALKEGWTRFKVKVGADLEDDIRRCRIIRDMIGPEKTLMVDANQRWDVPEAVEW-M

Choleopus -LCTGALKDGWTRFKVKVGADLQDDIRRCRLIRDMIGPHNTLMLDANQRWDVPEAVEW-M

Sorex -LCTKALKDGWTRFKVKVGGDIQDDIRRCRFIRNMIGPDKTLMLDANQRWDVPEAVEW-M

Cavia -LCAAALKDGWTRFKVKVGADLQDDMRRCRLIRDLIGPDRTLMMDANQRWDVPEAVAW-M

Loxodonta -LCSEALKEGWTRFKVKVGADLQDDIRRCRLIRDMIGPEKTLMMDANQRWDVHEAVEW-M

Bos -LCSEALKDGWTRFKVKVGADLQDDIRRCRLVRNMIGPEKTLMMDANQRWDVPEAVEW-M

Macaca -LCAQALKDGWTRFKVKVGADLQDDVRRCQIIRDMIGPEKTLMMDANQRWDVPEAVEW-M

Oryctolagus -RCAEALKDGWTRFKVKVGADLQDDMRRCRLIRDMIGPEKTLMMDANQRWDVPEAVEW-M

Equus WFCRQVLYSWWTLFKVKVGADLQDDIRRCRLIRNTIGPEKTLMMDANQRWDVPEAVEW-M

Ailuropoda -LCTEALKDGWTRFKVKVGADLQDDIRRCRLIRNMIGPEKTLMMDANQRWDVPEAIEW-M

Canis -LCTEALKAGWTRFKVKVGADLQDDVRRCRLIRNMIGPEKTLMMDANQRWDVPEAVKW-M

Otolemur -LCTEALKDGWTRFKVKVGADVQDDMRRCRLIRDMIGPENTLMMDANQRWDVPEAVEW-M

Callithrix -LCTQALKDGWTRFKVKVGADLQDDERRCQLIRDVIGPEKTLMMDANQRWDVPEAVEW-M

P.abelli -LCAQALKDGWTRFKVKVGADLQDDVRRCQIIRDMIGLEKTLMMDANQRWDVPEAVEW-M

P.pygmaeus -LCAQALKDGWTRFKVKVGADLQDDVRRCQIIRDMIGLEKTLMMDANQRWDVPEAVEW-M

Homo -LCAQALKDGWTRFKVKVGADLQDDMRRCQIIRDMIGPEKTLMMDANQRWDVPEAVEW-M

Gorilla -LCAQALKDGWTRFKVKVGADLQDDMRRCQIIRDMIGPEKTLMMDANQRWDVPEAVEW-M

Pan -LCAQALKDGWTRFKVKVGTDLQDDMRRCQIIRDMIGPEKTLMMDANQRWDVPEAVEW-M

Danio -LCNEALAQGWTKFKVKVGADLQDDIRRCSLIRKLIGPNNTLMIDANQRWDVNEAITW-V

Oryzias -LCTDALKGGWTKFKVKVGADLDDDVRRCRLIRQMIGPDNTLMIDANQRWDVSEAISW-V

Gasterosteus -LCTDALKSGWTRFKVKVGGDLEDDVRRCRLIRQMIGPENTLMIDANQRWGVAEAISW-V

Tetraodon -LCADALGNGWTKFKVKVGADLEDDRRRCRLIREMIGPSNTLMMDANQRWDVAEAIRW-V

Fugu -LCTDALQKGWTKFKVKVGADLEDDRRRCRLLRKIIGQSNTLMIDANQRWDVAEAIRW-V

. : : * . .** .*. : * * : * * : :: * :

FucD RQLAEFDIAWIEEPTSPDDVLGHAAIRQGITP--VPVSTGEHTQNRVVFKQLLQAGAVDL

C.intestinalis KQLVEFRPLWIEEPTCPDDVIGHATIAKALSPHNVGVATGEQCQNRVVFKQLLQVDGLKF

C.savignyi KPLVKFRPLWIEEPTSPDDVIGHATIAKALKEHKVGVATGEQCQNRVIFKQLMQTNAVSF

Rattus SILAEFKPLWIEKPTSQNDIIGHATISEALAPLGIGIATGEQCHNRVMIKQLLH-KALQL

Xenopus KDLAKYKPLWIEEPTSPDDILGHATISKALSPLNIGVATGEQCHNRVMFKQFLQANALQY

Anolis TKLAEFKPLWIEEPTSPDDVLGHATISKALALLGIGVATGEQCHNRVVFKQLLQAQALSY

Ornithorhynchus SKLAEFKPLWIEEPTSPDDILGHATISKALSPLGIGVATGEQCHNRVIFKQLLQAKALQY

Monodelphis LKLAEFKPLWIEEPTSPDDILGHATISKALAPLGIGVATGEQCHNRVIFKQLLQAGALQF

Choleopus LKLAEFKPLWIEEPTSPDDILGHAAIAK--------------CHNRVMFKQLLQAKALQF

Sorex AKLAEFKPLWIEEPTSPDDILGHAAISK--------------CHNRVMFKQLLQAKALQF

Cavia SKLAEFKPLWIEEPTSPDDILGHAAIAKALAPLGIGVATGEQCHNRVVFKQLLQANALQF

Loxodonta SQLAEFKPLWIEEPTSPDDILGHAAISKALAPLGIGVATGEQCHNRVIFKQLLQANALQF

Bos TKLAEFKPLWIEEPTSPDDILGHAAISKALAPLGIGVATGEQCHNRVIFKQLLQAKALKF

Macaca SKLAKFKPLWIEEPTSPDDILGHAAISKALVPLGIGIATGEQCHNRVIFKQLLQAKALQF

Oryctolagus SKLAEFKPLWIEEPTSPDDILGHATISKALVPLGIGVATGEQCHNRVIFKQLLQANALQF

Equus SKLAEFKPLWIEEPTSPDDILGHAAISKALVPLGIGVATGEQCHNRVIFKQLLQAKALQF

Ailuropoda SKLAEFKPLWIEEPTSPDDILGHATISKALAPLGIGVATGEQCHNRVIFKQLLQAKALQF

Canis SKLAEFKPLWIEEPTSPDDILGHATISKALAPLGIGVATGEQCHNRVIFKQLLQAKALQF

Otolemur SKLAEFKPLWIEEPTSPDDILGHAAISK--------------CHNRVIFKQLLQANALQF

Callithrix SKLAKFKPLWIEEPTSPDDILGHATISKALVPLGIGVATGEQCHNRVIFKQLLQVKALQF

P.abelli SKLAKFKPLWIEEPTSPDDILGHATISKALVPLGIGIATGEQCHNRVIFKQLLQAKALQF

P.pygmaeus SKLAKFKPLWIEEPTSPDDILGHATISKALVPLGIGIATGEQCHNRVIFKQLLQAKALQF

Homo SKLAKFKPLWIEEPTSPDDILGHATISKALVPLGIGIATGEQCHNRVIFKQLLQAKALQF

Gorilla SKLAKFKPLWIEEPTSPDDILGHATISRALVPLGIGIATGEQCHNRVIFKQLLQAKALQF

Pan SKLAKFKPLWIEEPTSPDDILGHATISKALVPLGIGIATGEQCHNRVIFKQLLQAKALQF

Danio TKLAEFQPLWIEEPTCPDDILGHASISKALAPLGIGVASGEQCHNRVMFKQFLQASALQF

Oryzias SNLAEVKPLWIEEPTSPDDILGHAAISKALAPLGIGVATGEQCHNRVMFKQFLQAGALQF

Gasterosteus SRLAEFKPLWIEEPTSPDDILGHAAISKALAPLGIGVATGEQCHSRVMFKQFLQASALQF

Tetraodon SSLADFKPLWIEEPTCPDDILGHAAISKALAPLGIGVASGEQCHNRVMFKQFLQASAL-F

Fugu SSLAEFNPLWIEEPTCPDDILGHAAISKALAPLGIGVASGEQCQNRVMFKQFLQASALQF

*.. ***:**. :*::***:* :.**::**::: .:

FucD IQIDAARVGGVNENLAILLLAAKFGVRVFPHAGGVGLCELVQHLAMADFVAITGKMEDRA

C.intestinalis LQIDSCRVGSINENIAILLMAAKFNVPVCPHAGGVGLCELVQHIIMFDYLCVSATNEQRM

C.savignyi VQIDSCRVGSINENIAILLMAAKFNLPVCPHAGGVGLCELVQHIIMFDYLCVSATTDGRV

Rattus LQIDSCRLGSVKENLSVSLMPKKFGIHLPPCWWSWTLNWLVQHLIIFDCQSLPAFKTGRM

Xenopus LQIDSCRLGSVNENLSVLLMSKKFNVPVCPHAGGVGLCELVQHLILFDYISVSGSLDNRM

Anolis VQIDSCRLGSVNENLSVLLMAKKFQIPVCPHAGGVGLCELVQHLIIFDYISISGSLENRM

Ornithorhynchus LQIDSCRLGSVNENLSVLLMAKKFQIPVCPHAGGVGLCELVQHLIIFDFISVSGSLENRM

Monodelphis LQIDSCRLGSVNENLSVLLMAKKFQIPVCPHAGGVGLCELVQHLIIFDYISISGSLENRM

Choleopus LQIDSCRLGSVNENLSVLLMAKKFGIPVCPHAGGVGLCELVQHLILFDYISVSACLQNRM

Sorex LQIDSCRLGSVNENLSVLLMAKKFQIPVCPHAGGVGLCELVQHLIIFDFISISASLENRM

Cavia LQIDSCRLGSVNENLSVILMAKKFGIPVCPHAGGVGLCELVQHLIIFDYISVSTSLQNRM

Loxodonta LQIDSCRLGSINENVSVLLMAQKFGIPVCPHAGGVGLCELVQHLIIFDYIAVSASLENRM

Bos LQIDSCRLGSVNENLSVLLMAKKFEIPVCPHAGGVGLCELVQHLIIFDFISVSASLQDRM

Macaca LQIDSCRLGSVNENLSVLLMAKKFEIPVCPHAGGVGLCELVQHLIIFDYISVSASLKNRM

Oryctolagus LQIDSCRLGSVNENLSVILMAKKFGIPVCPHAGGVGLCELVQHLIIFDYIAVSASLKNRM

Equus LQIDSCRLGSVNENLSVLLMAKKFEIPVCPHAGGVGLCELVQHLIIFDFISVSASLTNRM

Ailuropoda LQIDSCRLGSVNENLSVLLMAKKFEIPVCPHAGGVGLCELVQHLIIFDFISVSASLRNRM

Canis LQIDSCRLGSVNENLSVLLMAKKFEIPVCPHAGGVGLCELVQHLIIFDFISISASLQNRM

Otolemur LQIDSCRLGSVNENLSVLLMAKKFEIPVCPHAGGVGLCELVQHLIIFDYISVSASLENRM

Callithrix LQIDSCRLGSINENLSVLLMAKKFEIPVCPHAGGVGLCELVQHLIIFDYVSVSASLKNRM

P.abelli LQIDSCRLGSVNENLSVLLMAKKFEIPVCPHAGGVGLCELVQHLIIFDYISVSASLENRM

P.pygmaeus LQIDSCRLGSVNENLSVLLMAKKFEIPVCPHAGGVGLCELVQHLIIFDYISVSASLENRM

Homo LQIDSCRLGSVNENLSVLLMAKKFEIPVCPHAGGVGLCELVQHLIIFDYISVSASLENRV

Gorilla LQIDSCRLGSVNENLSVLLMAKKFEIPVCPHAGGVGLCELVQHLIIFDYISVSASLENRV

Pan LQIDSCRLGSVNENLSVLLMAKKFEIPVCPHAGGVGLCELVQHLIIFDYISVSASLENRV

Danio VQIDSCRVGSVNENLATILMAAKFNVPVCPHAGGVGLCELVQHLILFDYISVSASLSNRM

Oryzias VQIDSCRLGSVNENLAVLLMAHKFRVPVCPHAGGVGLCELVQHLILFDYICVSASLSNRM

Gasterosteus VQIDGCRVGSVNENLAVLLMAHKFQVPVCPHAGGVGLCELVQHLILFDYISVSGSLSNRM

Tetraodon VQIDSCRLGSVNENLAVLLMAHKFQVPVCPHAGGVGLCELVQHLILFDYICVSGSLANRM

Fugu VQIDSCRLGSINENLAVLLMAHKFQVPVCPHAGGVGLCELVQHLSLFDYICVSGSLTNRM

:***..*:*.::**:: *:. ** : : * . * ****: : * .:. *

FucD IEFVDHLHQHFLDPVRIQHGRYLAPEVPGFSAEMHPASIAEFSYPDGRFWVE--DLAASK

C.intestinalis CEYVDHLHEHFIEPVVIQNTCYMPPKKPGYSSEMKKDSVENYKFPDGKIWKD--LIEGGK

C.savignyi CEYVDHLHEHFVEPVRIKDASYMSPQKPGYSSEMKAESLDNYEFPNGTIWSN--LIKEGK

Rattus CEYVDHLHEHFKYPMVIKHASYLPPKDAGFSTKMK-------------------------

Xenopus CEYVDHLHEHFMYPVIISRAAYMPPKDPGYSTEMKDESVLQYQFPDGEIWQK--------

Anolis CEYVDHLHEHFKYPVVIKNASYMPPQAPGYSSEMKEDSVRKYQFPQGEIWQK--LLSDPQ

Ornithorhynchus CEFVDHLHEHFKYPVVIKKASYMPPQDPGYSSEMKEDSVKQHEFPEGEVWQN--LMAAQK

Monodelphis CEYVDHLHEHFKYPVVIKKASYMPPKDAGYSTEMKEESVKEHQFPEGKIWQK--LMAAQK

Choleopus CEYVDHLHEHFKYPVTIKKASYMPPKDAGYSTEMKEESVNEYQYPDGEVWKK--LLAAQE

Sorex CEYVDHLHEHFKYPVRIQKAAYMPPKDPGYSTEMKEESIKQHQYPDGDVWKK--LLAAQG

Cavia CEYVDHLHEHFKYPVYIKQASYMPPQDAGYSTEMKEDSVKKHQYPDGEVWRK--LLAAQE

Loxodonta CEYVDHLHEHFKYPVIIKKASYMPPEDPGYSTEMKEESVKKHQFPDGEVWTK--LLAGQE

Bos CEYVDHLHEHFKYPVLIREAAYMPPKDAGYSTEMKEDSVKRHRYPDGEVWKK--LLSAQG

Macaca CEYVDHLHEHFKYPVMIQRASYMPPKDPGYSTEMKEESVKKHQYPDGEVWKK--LLAAQE

Oryctolagus CEYVDHLHEHFKYPVTIKQASYMPPKAAGYSTEMKEESVKKHQYPEGEVWKR--LHAAHE

Equus CEYVDHLHEHFKYPVVIEKASYMPPKDAGYSTEMKEDSVKKHQYPDGEVWKK--LLAARG

Ailuropoda CEYVNHLHEHFRYPVIIKKASYMPPKDAGYSTEMKEESIKKHQYPDGEVWKK--LLAAQE

Canis CEYVDHLHEHFRYPVIIKKASYMPPKDAGYSTEMKEESVKKHQYPDGEVWKK--LLAAQE

Otolemur CEYVDHLHEHFKYPVKIRQASYMPPMDAGYSTEMKEESVKKHQYPDGEVWKK--LLATQE

Callithrix CEYVDHLHEHFKYPVVIQRASYMPPKDAGYSTEMKEESVKKHQYPDGEVWKK--LLAAQE

P.abelli CEYVDHLHEHFKYPVMIQRASYMPPKDPGYSTEMKEESVKKHQYPDGEVWKK--LLAAQE

P.pygmaeus CEYVDHLHEHFKYPVMIQRASYMPPKDPGYSTEMKEESVKKHQYPDGEVWKK--LLAAQE

Homo CEYVDHLHEHFKYPVMIQRASYMPPKDPGYSTEMKEESVKKHQYPDGEVWKK--LLPAQE

Gorilla CEYVDHLHEHFKYPVMIQQASYMPPKDPGYSTEMKEESVKKHQYPDGEVWKK--LLPAQE

Pan CEYVDHLHEHFKYPVMIQRASYMPPKDPGYSTEMKEESVKKHQYPDGEVWKK--LLPAQE

Danio CEFVDHLHEHFKSPTVIRNAKYIPPKDPGFSCEMLEESVKKHQYPEGEVWRA--IEKQQK

Oryzias CEYVDHLHEHFVCPVVIHNARYMPPKIPGYSCEMLESSVKKHQYPDGDAWK---LYPKK-

Gasterosteus CEYVDHLHEHFTSPVVIRDAHYMPPKDPGFSCEMLESSVQRHQYPEGEVWKL--NISK--

Tetraodon CEYVDHLHEHFASPVVIRNGHYIAPEDIGYSCEMLESSVQSHRYPEGDVWKA--NTSKPE

Fugu CEYVDHLHEHFASPVVIRNGHYMPPKDLGYSCEMLASSVQAHRYPEGDVWKKTFLVVSSG

*:*:***:** * * *:.* *:* :*

FucD AKA---

C.intestinalis FVQ---

C.savignyi FKM---

Rattus ------

Xenopus ------

Anolis T-----

Ornithorhynchus L-----

Monodelphis N-----

Choleopus N-----

Sorex N-----

Cavia N-----

Loxodonta N-----

Bos N-----

Macaca N-----

Oryctolagus N-----

Equus N-----

Ailuropoda N-----

Canis N-----

Otolemur N-----

Callithrix N-----

P.abelli N-----

P.pygmaeus N-----

Homo N-----

Gorilla N-----

Pan N-----

Danio ------

Oryzias ------

Gasterosteus ------

Tetraodon VKMDTF

Fugu N-----
