# Supplementary material for: Transient knockdown and overexpression reveal a developmental role for the zebrafish enosf1b gene
Source: Cell Biosci. 2011 Sep 26;1:32. doi: 10.1186/2045-3701-1-32 (PMC3197473; doi:10.1186/2045-3701-1-32)
Supplement: Additional file 3 — Protein sequences of predicted ENOSF1βs with missing exons and not used in phylogenetic analysis. Ensembl accession numbers follow the genus name. [file 2045-3701-1-32-S3.DOC]

**Additional file 3: Protein sequences of predicted ENOSF1βs with missing exons and not used in phylogenetic analysis.**

Ensembl accession numbers follow the genus name.

>Macropus ENSMEUP00000008687

SDAMVRAGHTDPDYSAAYVIIETDAKDGLKGHGITFTLGKGTEVVVCAVNALSHHVLNKD

LGDIVRDFRGFYRQLTSDGQLRWIGPEKGVVHLATAAVLNALWDLWAKQEGKPLWKLLVD

MDPRQLLSCIDFRYITDALTEEEAYEILQKGSIGRKDRXXXXXXXXXXXXXXXXXXXXXX

XXXXXXXXXXXXXXXXXXFKVKVGADLEDDIRRCRLIRDMIGPEKILXXXXXXXXXXXXX

XXXXXXXXXXXXXXXXXXXXXXXXXXXXXXXXCHNRVIFKQLLQAGALQFLQIDSCRLGS

VNENLSVLLMAKKFQIPVCPHAGGVGLCELVQHLIIFDYISVSGSLENRMCEYVAHLHEH

FKYPVVIKNASYMPPK

>Microcebus ENSMICP00000013535

HTDPDYSAAYVVIETDADDGLKGYGITFTLGKGTEVVVCAVNALAHHVLNKDLRDIVGDF

RGFYRQLTSDGQLRWIGPEKGVVHLATAAVLNAVWDLWAKQEGKPLWKLLVDMDPRTLVS

CIDFRYITDVLTEEDAYEILQKAQVGKKEREEHMLTRGYPAYTTSCAWLGYSDDTLKQLC

KEALRDGWTRFKVKVGADVQDDVRRCRLIRDMIGPDKTLXXXXXXXXXXXXXXXXXXXXX

XXXXXXXXEPTSPDDILGHAAISKCHNRVIFKQLLQANALQFLQIDSCRLGSVNENLSVL

LMAKKFEIPVCPHAGGVGLCELVQHLIIFDYISVSASLENRMCEYVDHLHEHFKYPVRIR

QASYMPPMDAGYSTEMKEESVKKHQYPDGEVWKKLLAAQKN

>Tupaia ENSTBEP00000000674

VCAVNALAHHVLNKDLRDIVGDFRGFYRQLTSDGQLRWIGPEKGVVHLATAAVLNAVWDL

WAKQEGKPLWRLLVDMDPRTLLSCIDFRYITDVLTEEEAYEILQKGQLGKKEREEQMLTH

GYPAYTTSCAWLGYSDDTLKQLCTEALKDGWTRFKVKVGADLKDDMRRCRLIRDMIGPEK

ALXXXXXXXXXXXXXXXXXXXXXXXXXXXXXXXXXXXXXXXXXXXXXCHNRVIFKQLLQA

NALQFLQIDSCRLGSVNENLSVLLMAKKFEIPVCPHAGGVGLCELVQHLIIFDYISVSTS

LQNRMCEYVDHLHEHFKYPVTIKQASYMPPQ

>Felis ENSFCAP00000008558

HTDPDYSAAYVVLETDVEDGLKGYGITFTLGKGTEVVACAVNALAHHVLNKDLGDIVGDF

RGFYRQLTSDGQLRWIGPEKGVVHLATAAVLNAVWDLWAKQEGKPLWKLLVDMXXXXXXX

XXXXXXXXXXXXXXXXXXILQKGQIGKKERERQMLMHGYPAYTTSCAWLGYSDDTLKQLC

TEALQDGWTRFKVKVGADLQDDIRRCRLIRNMIGPEKTLXXXXXXXXXXXXXXXXXXXXX

XXXXXXXXXXXXXXXXXXXXXXXXCHNRVIFKQLLQAKALQFLQIDSCRLGSVNENLSVL

LMAKKFEXXXXXXXXXXXXXXXXXXXXXXXXXXXXXXXKNRMCEYVDHLHEHFRYPVTIK

KASYMPPKDAGYSTEMKE

>Squirrel ENSSTOP00000013920

HTDPDYSSAYVVLETDVGDGLKGYGITFTLGKGTEVVVCAVNALAHHVLHKDLKDIVSDF

RGFYRQLTSDGQLRWIGPEKGVVHLATAAILNAVWDLCAKQEGKPLWKLLVDMDPRALLS

CIDFRYITDVLTEEDAYEILKKGQIGKKEREEQMLMXXXXXXXXXXXXXXXXXXXXXXLC

AAALKDGWTRFKVKVGADLQDDMRRCHLIRDMIGPEKTLMMDANQRWDVPSKLAEFKPLW

IEEPTSPDDILGHAAISKCHNRVIFKQLLQVNALQFLQIDSCRLGSVNENLSVLLMAKKF

GIPVCPHAGGVGLCELVQHLIIFDYISVSASLKN

>Squirrel ENSSTOP00000012210

SDAMVSLTDASGARDNHTDPDYSSAYVVLETDVGDGLKGYGITFTLEKGTEVVVCAVNAL

AHHVLHKDLKDIVSDFRGFYRQLTSDGQLRWIGPEKGVVHLATAAILNAVWDLWANQEGK

PLWKLLVDMDPRALLSCIDLRYITDVLTEEDAYEILKKGQTGKKERKEQVLMHGYPAYTT

SCAWLGYSDDTLKQLCAAALKDGTXXXXXXXXXXXXXXXXXXXXXDMIGPEKTLMMDANQ

HWDVPEAVEWMSKWIEEPTSPDDILGHAAISKCHNRVIFKQLLQANALQFLQIDSCRLGS

VNENLSVLLMAKKFGIPVCPHAGGVGLCELVQHLIIFDYISVSASLKNRMCEYVDHLHEH

FKYPVVIKQASYMPPKDAGYSTEMKEDSVRKHQYPDGDVWKKLLAAQEN

>Dipodomys ENSDORP00000014189

SDAMVSTDXXXXXXXXXXXXXXXXXXXXXXXXXXXXXXXXXXXXXXXXXXXXXXXXXXXX

XXXXXXXXXXXXXXXXXXXXXXXXXXXXXXXXXXXXXXXXXXXXXXXXXXXXXXXXXXXX

XDPRMLLSCIDFRYITDVLTEEEAYEILQKGQVGKKEREKQMLTHGYPAYTTSCAWLGYS

DTTLKQLCAAALKDGWTRFKVKVGADLQDDIRRCRLIRDMIGPEKILMMDANQRWDVPEA

VEWMSKLAEFKPLWIEEPTSPDDILGHATISKCHNRVIFKQLLQADALQFLQIDSCRLGS

VNENLSVLLMAKKFGIPVCPHAGGVGLCELVQHLIIFDFISISGSLKNRMCEYVDHLHEH

FKYPVIIKQASYMPPAAGYSTEMKEESVQKHQYPHGEVWKKLLPAQED

>Ochotona ENSOPRP00000005496

SDALVSADHTDPDYSAAYVVIETDAEDGLRGCGITFTLGKGTEVVVCCVNALAQHVLNKD

FKDIIGNFRNFYRQLTSDGQLRWIGPEKGVVHLATAAILNAIWDLWAKQEGKPLWKMLVD

MXXXXXXXXXXXXXXXXXXXXXXXXXXXXXXXXXXXXXXXXXXXXXXXXXXXXXXXXXXX

XXXXXXRCAEALKDGWTXXXXXXXXXXXXXXXXXXXXXXXXXXXXXXMMDANQRWDVPEA

VEWMLALAEFKPLWIEEPTSPDDILGHAAISKCHNRVIFKQLLQANALQFLQIDSCRLGS

VNENVSVLLMAKKFGIPVCPHAGGVGLCELVQHLIMFDYIAVSGSLKNRMCEYVDELHEH

FKYPVTIKDASYMPPKAPGYSTEMKEDSVEKHRFPIGEVWQRLLAAHEN

>Tarsius ENSTSYP00000010774

VCAVTALTHHVINKDLRDIVSDFRGFYRRLTSDGQLRWIGPEKGVVHLATAAILNAVWDL

WAKQEGKPLWKLLVDMDPRTLVSCIDFRYITDVLTEEDAYEILQKGQVGKKEREEQMQTH

GYPAYTTSCAWLGYSDDMLKQLCTEALKDGWTXXXXXXXXXXXXXXXXXXXXXXXXXXXX

XXMMDANQRWDVPEAVEWTSRLAEFKPLWIEEPTSPDDILGHAAISKCHNRVIFKQLLQA

KALQFLQIDSCRLGSVNENLSVLLMAKKFEIPVCPHAGGVGLCELVQHLIIFDYISVSAS

LKNRMCEYVDHLHEHFKYPVVIQQASYMPPKEAGYSTEMKEESVQKHQYPDGEVWKKLLA

SQEN

>Dasypus ENSDNOP00000013759

SDAMVSAGDHTDPDYSAAYVVIETDAKDGLKGCGLTFTLGKGTEVVACAVNALAHHLLHK

DIRDIVSDFRSFYRQLTSDGQLRWXXXXXXXXXXXXXXXXXXXXXXXXXXXXXXXXXXXX

XXDPKTLLSCIDFRYITDVLTEEEAYEILRKGXXXXXXXXXXXXXXXXXXXXXXXXXXXX

XXXXXXXXXXXXXXXXXXXFKVKVGADLQDDVRRCCLIRDMIGPENTLMLDANQRWDVPE

AVEWTQKLATFKPLWIEEPTSPDDILGHAAIAKCHNRVIFKQLLQAKALQFLQIDSCRLG

SVNENLSVLLMAKKFEIPVCPHAGGVGLCELVQHLILFDYISVSASLQNRMCEYVDHLHE

HFEYPVTIKKAAYMPPKDAGYSTEMKEESIKKYQYPDGEVWKKLLAAQEY

>Myotis ENSMLUP00000008762

SDAMVSADHTDPDYSAAYVVLETDAEDGLRGHGITFTLGRGTEVVVCAVNALAHHVLNKD

LEDIVGDFRGFYRQLTSDGQLRWXXXXXXXXXXXXXXXXXXXXXXXXXXXXXPLWKLLVD

MDPRTLLSCIDFRYITDVLTEEEAYEILQEGQVGKKEREEQMLTLGYPAYTTSCAWLGYS

DDMLKQLCTEALKGGWTRFKVKVGADLQDDIRRCRLIRNMIGPEKTLMMDANQRWDVPEA

VEWMAKLAEFKPLWIEEPTSPDDILGHATISKCHNRVMFKQLLQAKALQFLQIDSCRLGS

VNENLSVLLMAKKFEIPVCPHAGGVGLCELVQHLILFDFISVSASLTNRMCEYVDHLHEH

FKYPVTIKNASYMLPKEAGYSTEMKEESIKKHQYPDGEVWKKLLTAQGN

>Pteropus ENSPVAP00000000665

SDAVVSAATSVPSHTDPDYSAAYVVLETDAEDGLEAYGITFTLGKGTEVVVCAVNALAHH

VLNEDLRDIVGNFRGFYRQLTSDGQLRWIGPKKGVVHLATAAILNAVWDLWAKQEGKXXX

XXXXXXDPRMLLCIDFRYITEVLTEEEAYEILQKGQKEREGQMLTHGYPAYTTTCAWLGY

SDDTLKQLCTEALMDGWTRFKVKVGANLRDDIHRCRLIRNMIGPEKTLMMNANQCWDVPE

AVEWMSNLAEFKPLWIEEPTSPDDVLGHATISKCNNRVILKQLLQAKALQFLQIDSCRLG

SVNENLSVLPMAKKLEIPVCPHAGGVGLCELVQHLILFDFISVSASLKNRMCEYVDYLHE

HFKYPVIIKNASYMPPQDAGYSTEMKEESVKKYQYPDGEVWKKLLTAKGN

>Tursiops ENSTTRP00000015805

SDAMVSAATAGAHTDPDYSAAYVVLETDAEDGLKGYGITFTLGKGTEVXXXXXXXXXXXX

XXXXXXXXXXXXXXXXXXXXXXXXXXXIGPEKGVVHLATSAVLNAVWDLWAKQEGKPLWK

LLVDMDPRKLVSCLDFRYITDVLTEEEAYEILQKGQVGKKEREEQMLAHGYPAYTTSCAW

LGYSDTTLKQLCSKALEDGWTRFKVKVGADLQDDIRRCRLIRNMIGPEKTLMMDANQRWD

VPEAVEWMAKLAEFKPLWIEEPTSPDDILGHATISKCHNRVIFKQLLQAKALQFLQIDSC

RLGSVNENLSVLLMAKKFDIPVCPHAGGVGLCELVQHLIIFDFISVSASLKDRMCEYVDH

LHEHFKYPVIIKKASYMPPKDAGYSTEMKEESVKKHQYPDGEVWEKLLAAQGN

>Erinaceus ENSEEUP00000008294

SDAMVSHTDPDYSTAYVTLETDAEDGLKGYGITFTLGKGTEVXXXXXXXXXXXXXXXXXX

XXXXXXXXXXXXXXXXXXXXXIGPEKGVVHLATAAILNAVWDLWAKQEGKPLWKLLVDMD

PRTLSSCIDFRYISDVLTEEEACEILQKNQAGKKEREAQMLTQGYPAYXXXXXXXXXXXX

XXXXLCTEALKGGWTRFKVKVGADMQDDVRRCGLIRDMIGPEKTLMMDANQRWDVPEAVE

WMARLAEFKPLWIEEPTSPDDILGHAAISRCHNRVIFKQLLQARALQFLQIDSCRLGSVN

ENLSVLLMAKKFGIPVCPHAGGVGLCELVQHLIIFDFISVSASLENRMCEYVDHLHEHFK

YPVLIQKACYLPPK

>Procavia ENSPCAP00000015174

SDATVSTAGFGAGGTGALHTDPDYSAAYVIIETDAEDGLKGYGITFTLGRGTEVXXXXXX

XXXXXXXXXXXXXXXXXXXXXXXXXXXXXXXXXIGPEKGVIHLATAAILNALWDLWAKQE

GKPLWKLLVDMXXXXXXXXXXXXXXXXXXXXXXXXXXXXXXXXXXXXXXAQMLAHGYPAY

TTSCAWLGYSDDTLKELCSQALKDGWTRFKVKVGADLQDDIRRCRLIRHMIGPEKTLMMD

ANQRWDVPEAVEWMSKLAEFNPLWIEEPTSPDDILGHAAISKCHNRVMFKQLLQANALQF

LQIDSCRLGSVNENLSVLLMAQKFGIPVCPHAGGVGLCELVQHLIIFDYIAISASLKNRM

CEYVDHLHEHFKYPVIIQKASYMPPKDAGYSTEMKEESVKKHQFPDGEVWKKLLAGQEN

>Echinops ENSETEP00000013550

SDAMVSDAGDAAXXXXXXXXXXXXXXXXXXXXXXXXXXXXXXXXXXXXXVCAVKALAHHV

LNKALGDIVGDFRGFYRQLTSDGQLRWIGPEKGVVHLATAAVLNALWDLWAKQEGKPLWK

LLVDMXXXXXXXXXXXXXXXXXXXXXXXXXXXXXXXXXXXXXXXXXXXXXXXXXXXXXXX

XXXXXXXXXXFCSKSGGDGRAXXXXXXXXXXXXXXXXXXXXXXXXXXXXXMMDANQRWDV

PEAVEWMVKLAEFKPLWIEEPTSPDDILGHATISKCHNRVMFKQLLQAKALQFLQIDSCR

LGSVNESLSVLLMAHKFGIPVCPHAGGVGLCELVQHLIIFDYIAVSASLENRMCEYVDHL

HEHFKYPVLIQKASYMPPKDAGYSTEMKESSVKEHQFPGGAVWKKLLAGREN
