# Supplementary material for: Transient knockdown and overexpression reveal a developmental role for the zebrafish enosf1b gene
Source: Cell Biosci. 2011 Sep 26;1:32. doi: 10.1186/2045-3701-1-32 (PMC3197473; doi:10.1186/2045-3701-1-32)
Supplement: Additional file 4 — E10i10-injected embryos express enosf1b transcript with inserted intron 10. Sequence of mis-spliced product generated from e10i10-injected embryo cDNA and PCR with exon 10 flanking primers. Exons and introns are color-coded. [file 2045-3701-1-32-S4.DOC]

**Additional file 4: E10i10-injected embryos express *enosf1b* transcript with inserted intron 10.**

Sequence of mis-spliced product generated from e10i10-injected embryo cDNA and PCR with exon 10 flanking primers. Exons and introns are color-coded.

CTGCCTATACCACTTCCTGTGCCTGGCTGGGTTACACTGACCARCAGCTTACACAGCTCTGCAATGAASCTCTTGCTCAAGGATGGACTAAATTTAAAGTGAAAGTCGGGGCTGATTTGCAGGATGATATTCGTAGATGCAGCCTTATTCGAAAGCTGATTGGACCAAACAACACACTGATGATTGATGCCAACCAACGGTGGGATGTTAATGAAGCAATCACCTGGGTAACCAAGCTGGCAGAGTTTCAGCCACTGTGGATTGAGGAGCCCACCTGTCCTGATGACATTCTAGGTCATGCTTCCATCTCTAAGGTAAACTGGTTCAATATGTTCACTATAATCTCATGCAAGTCAGGTTTTGAGTTTTTGTGTCTGTGTGGTGTTCTAGGCACTTGCACCGTTGGGTATCGGAGTTGCCTCTGGAGAGCAGTGCCATAACAGAGTGATGTTCAAGCAGTTTCTCCAGGCCTCAGCTCTGCA

Exon 10

Intron 10

Exon 11
