# Supplementary material for: Transient knockdown and overexpression reveal a developmental role for the zebrafish enosf1b gene
Source: Cell Biosci. 2011 Sep 26;1:32. doi: 10.1186/2045-3701-1-32 (PMC3197473; doi:10.1186/2045-3701-1-32)

**Additional file 5: Alignments of conserved regions targeted by morpholino antisense oligonucleotides used in this study.**

Alignment done in MUSCLE.


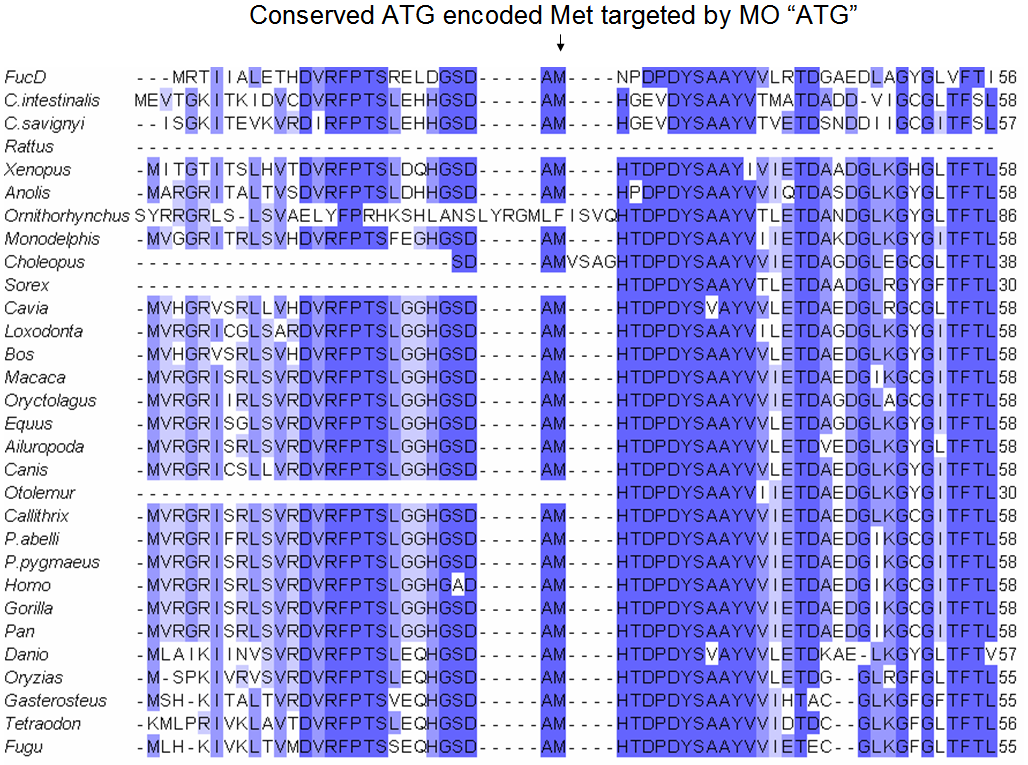


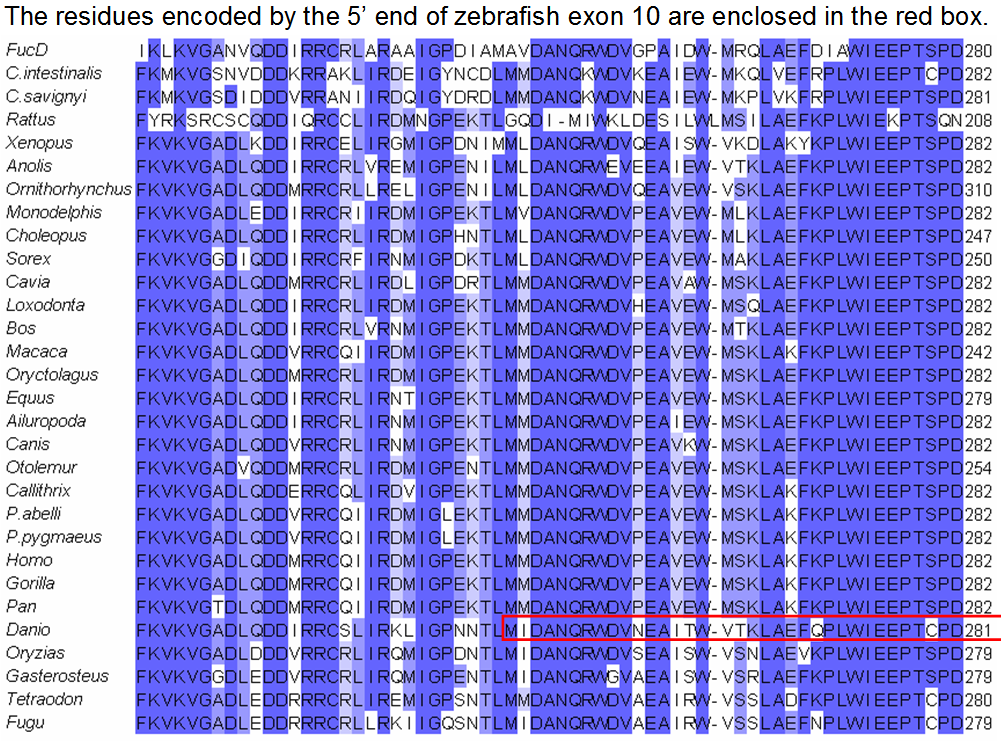

Supplement: Additional file 5 — Alignments of conserved regions targeted by morpholino antisense oligonucleotides used in this study. Alignment done in MUSCLE. [file 2045-3701-1-32-S5.DOC]
