# Supplementary material for: Transient knockdown and overexpression reveal a developmental role for the zebrafish enosf1b gene
Source: Cell Biosci. 2011 Sep 26;1:32. doi: 10.1186/2045-3701-1-32 (PMC3197473; doi:10.1186/2045-3701-1-32)
Supplement: Additional file 6 — Creating the enosf1b WISH plasmid. Sequences of enosf1b in plasmids p-enosf1b (Additional figure 1) and pBS-enosf1b-5' (Additional figure 2). See Methods for rationale. [file 2045-3701-1-32-S6.DOC]

**Additional File 6: Creating the *enosf1b* WISH plasmid*.***

Sequences of *enosf1b* in plasmids p-*enosf1b* (Additional figure 1) and pBS-*enosf1b*-5' (Additional figure 2). See Methods for rationale.

**Additional figure 1: Alignment of p-*enosf1b* insert to transcript encoding predicted drENOSF1β full length sequence.**

Note that the poorly aligned sequence starts at position 1015 and continues to 1282 before terminating in a polyA tail. Alignment done in MUSCLE.

*p-enosf1b* GTCTTTGGGCGGAGCGTGTGAGTTGTACTGTAGTATTGGATTACAGTCGAGTGTACAGTA 60

*enosf1b*  GTCTTTGGGCGGAGCGTGTGAGTTGTATAGTAGTATTGGATTACAGTCGAGTGTACAGTA 60

*************************** *******************************

*p-enosf1b* GGTGGAGTTTACAAAATGCTGGCGATCAAAATCATAAATGTGTCCGTGCGTGATGTGAGA 120

*enosf1b*  GGTGAAGTTTACAAAATGCTGGCGATCAAAATCATAAATGTGTCCGTGCGTGATGTGAGA 120

**** *******************************************************

*p-enosf1b* TTCCCGACGTCACTGGAACAACATGGATCCGATGCAATGCACACAGATCCAGATTATTCT 180

*enosf1b*  TTCCCGACGTCACTGGAACAACATGGATCCGATGCAATGCACACAGATCCAGATTATTCT 180

************************************************************

*p-enosf1b* GTGGCGTATGTTGTTCTGGAGACGGACAAAGCTGAACTCAAGGGTTACGGCTTGACTTTT 240

*enosf1b*  GTGGCGTATGTTGTTCTGGAGACGGACAAAGCTGAACTCAAGGGTTACGGCTTGACTTTT 240

************************************************************

*p-enosf1b* ACTGTGGGAAGAGGGACGGAAATCGTTGTGTGTGCAGTTAAAGCTTTATCCACCCTGGTG 300

*enosf1b*  ACTGTGGGAAGAGGGACGGAAATCGTTGTGTGTGCAGTTAAAGCTTTATCCACCCTGGTG 300

************************************************************

*p-enosf1b* GTGGGGAAGACTTTGGAGGAAATAACCAGTGACTTCCGAGGCTTCTACAGACTCCTGAGC 360

*enosf1b*  GTGGGGAAGACTTTAGAGGAAATAACCAGTGACTTCCGAGGCTTCTACAGACTCCTGAGC 360

************** *********************************************

*p-enosf1b* AGCGATGGTCAGATGAGATGGATCGGACCAGAGAAAGGAGTCATTCATCTGGCAACAGCA 420

*enosf1b*  AGCGATGGTCAGATGAGATGGATCGGACCAGAGAAAGGAGTCATTCATCTGGCAACAGCA 420

************************************************************

*p-enosf1b* GCCGTATTGAATGCTGTCTGGGATTTATGGGCAAGAGTAGAGAGAAAGCCCTTGTGGAAG 480

*enosf1b*  GCCGTATTGAATGCTGTCTGGGATTTATGGGCAAGAGTAGAGAGAAAGCCCTTGTGGAAG 480

************************************************************

*p-enosf1b* CTACTTGTTGACATGGATCCTGCAAAGCTGATCAGCTGTATTGATTTCAGATATCTCACT 540

*enosf1b*  CTACTTGTTGACATGGATCCTGCAAAGCTGATCAGCTGTATTGATTTCAGATATCTCACT 540

************************************************************

*p-enosf1b* GATGCTCTGACGGAGCAGGAAGCTTTAGATATATTGGTAAAAGGCAAAAAAGATCAGAAA 600

*enosf1b*  GATGCTCTGACGGAGCAGGAAGCTTTAGATATATTGGTAAAAGGCAAAAAAGATCAGAAA 600

************************************************************

*p-enosf1b* AGTAGAGAGGAACAGATGTTGAAAGAAGGCTATCCTGCCTATACCACTTCCTGTGCCTGG 660

*enosf1b*  AGTAGAGAGGAACAGATGTTGAAAGAAGGCTATCCTGCCTATACCACTTCCTGTGCCTGG 660

************************************************************

*p-enosf1b* CTGGGTTACACTGACCAGCAGCTTACACAGCTCTGCAATGAAGCTCTTGCTCAAGGATGG 720

*enosf1b*  CTGGGTTACACTGACCAGCAGCTTACACAGCTCTGCAATGAAGCTCTTGCTCAAGGATGG 720

************************************************************

*p-enosf1b* ACTAAATTTAAAGTGAAAGTCGGGGCTGATTTGCAGGATGATATTCGTAGATGCAGCCTT 780

*enosf1b*  ACTAAATTTAAAGTGAAAGTCGGGGCTGATTTGCAGGATGATATTCGTAGATGCAGCCTT 780

************************************************************

*p-enosf1b* ATTCGAAAGCTGATTGGACCAAACAACACACTGATGATTGATGCCAACCAACGGTGGGAT 840

*enosf1b*  ATTCGAAAGCTGATTGGACCAAACAACACACTGATGATTGATGCCAACCAACGGTGGGAT 840

************************************************************

*p-enosf1b* GTTAATGAAGCAATCACCTGGGTAACCAAGCTGGCAGAGTTTCAGCCACTGTGGATTGAG 900

*enosf1b*  GTTAATGAAGCAATCACCTGGGTAACCAAGCTGGCAGAGTTTCAGCCACTGTGGATTGAG 900

************************************************************

*p-enosf1b* GAGCCCACCTGTCCTGATGACATTCTAGGTCATGCTTCCATCTCTAAGGCACTTGCACCG 960

*enosf1b*  GAGCCCACCTGTCCTGATGACATTCTAGGTCATGCTTCCATCTCTAAGGCACTTGCACCT 960

***********************************************************

*p-enosf1b* TTGGGTATCGGAGTTGCCTCTGGAGAGCAGCACCA-GATATAGCTGTGGTCAGGGG---- 1015

*enosf1b*  TTGGGTATCGGAGTTGCCTCTGGAGAGCAGTGCCATAACAGAGTGATGTTCAAGCAGTTT 1020

****************************** *** * * ** ** *** *

*p-enosf1b* ----------CAAATGTGAATATTAG--AGGGTGGGGTTTGGGGGGATGGGGGTCATG-- 1061

*enosf1b*  CTCCAGGCCTCAGCTCTGCAGTTTGTTCAGATTGACAGCTGTAGGGTCGGCAGTGTTAAT 1080

** * ** * ** ** ** ** *** ** ** *

*p-enosf1b* --GGCCCCTAATAATATGTCTGGGGCTCCAAAAATGCATGGGCCCTGAGA-----ATTGT 1114

*enosf1b*  GAGAACCTTGCCACTATACTCATGGCTGCCAAGTTTAATGTTCCTGTATGTCCTCATGCT 1140

* ** * * *** **** * ** * *** ** * ** *

*p-enosf1b* CCAAACTTTAGCCCCCAC---CCCACCCACCTCCACCACCCCTGCAGCGGCACCCC--TG 1169

*enosf1b*  GGCGGTGTTGGACTGTGTGAGCTTGTACAGCACCTTATTTTGTTTGACTACATTTCAGTG 1200

** * * * ** * ** * * ** * **

*p-enosf1b* GCTG-TGGTGTACACAACAGTTGATCCCAGTT---GTGTTGTCC-CATTTCCATCTGGAC 1224

*enosf1b*  TCTGCTAGTTTAAGTAATAGGATGTGTGAGTTCGTGGATCACCTTCATGAACACTTCAAA 1260

*** * ** ** ** ** * **** * * * *** ** * *

*p-enosf1b* ACAGACATTTT--TTTAAAATAATGGGTGCATGGAAGCACAACTTTCTGAAAAAAAAAAA 1282

*enosf1b*  AGCCCCACCGTGATCAGAAATGCCAAATACATTCCACCAAAAGACCCTGGATTCTCTTGT 1320

* ** * * **** * *** * ** ** *** *

*p-enosf1b* AAAAA------------------------------------------------------- 1287

*enosf1b*  GAAATGCTGGAGGAATCTGTGAAAAAGCATCAGTATCCTGAAGGGGAGGTTTGGAGAGCC 1380

***

*p-enosf1b* ------------------------------------------------------------

*enosf1b*  ATTGAGAAACAGCAGAAGTGATAGAAACCAACACAAGCACTGACTGCTGAGGAAATTAAA 1440

*p-enosf1b* ------------------------------------------------------------

*enosf1b*  AATGACCAACGAAAGGGAGAAAAATACTTAAAGACCTGTCAGGTTGTCCTGATTGACTTA 1500

*p-enosf1b* ------------------------------------------------------------

*enosf1b*  AAGGGCATTATAAATGGATGGGAGAGTAAATAGGGAAATATATATTTTCTTAAACAAATG 1560

*p-enosf1b* ------------------------------------------------------------

*enosf1b*  GTTTAAACTATATGGAGTCTTTACTGTAAGTCTTTACTCTGTTTTGTCTTATTTCAATGT 1620

*p-enosf1b* ------------------------------------------------------------

*enosf1b*  TCTTAAAGGTCATACTGCACTGTGACAGGACTAGAGATCAAGTGTATGCTGACACTATTT 1680

*p-enosf1b* ------------------------------------------------------------

*enosf1b*  GTTTAATAGTCTGAATAATGACTAAAATGGGGAATTTTATCTGTGCATTAACTGAAGGGG 1740

*p-enosf1b* ------------------------------------------------------------

*enosf1b*  ACTGTATGGTATATGTACTTGGTGAATGTTACCAATCAAACTGAAAAAAACTTTTCTATT 1800

*p-enosf1b* ---------------------------------------

*enosf1b*  TTTATGCATTTCCTGACATAAATATGTAATGTTTTGGTC 1839

**Additional figure 2: XhoI-EcoRV *enosf1b* fragment of pBS-*enosf1b*-5' aligned to transcript encoding predicted drENOSF1β full length sequence.**

Alignment done in MUSCLE

XhoIEcoRV GTCTTTGGGCGGAGCGTGTGAGTTGTACTGTAGTATTGGATTACAGTCGAGTGTACAGTA 60

*enosf1b*  GTCTTTGGGCGGAGCGTGTGAGTTGTATAGTAGTATTGGATTACAGTCGAGTGTACAGTA 60

*************************** *******************************

XhoIEcoRV GGTGGAGTTTACAAAATGCTGGCGATCAAAATCATAAATGTGTCCGTGCGTGATGTGAGA 120

*enosf1b*  GGTGAAGTTTACAAAATGCTGGCGATCAAAATCATAAATGTGTCCGTGCGTGATGTGAGA 120

**** *******************************************************

XhoIEcoRV TTCCCGACGTCACTGGAACAACATGGATCCGATGCAATGCACACAGATCCAGATTATTCT 180

*enosf1b*  TTCCCGACGTCACTGGAACAACATGGATCCGATGCAATGCACACAGATCCAGATTATTCT 180

************************************************************

XhoIEcoRV GTGGCGTATGTTGTTCTGGAGACGGACAAAGCTGAACTCAAGGGTTACGGCTTGACTTTT 240

*enosf1b*  GTGGCGTATGTTGTTCTGGAGACGGACAAAGCTGAACTCAAGGGTTACGGCTTGACTTTT 240

************************************************************

XhoIEcoRV ACTGTGGGAAGAGGGACGGAAATCGTTGTGTGTGCAGTTAAAGCTTTATCCACCCTGGTG 300

*enosf1b*  ACTGTGGGAAGAGGGACGGAAATCGTTGTGTGTGCAGTTAAAGCTTTATCCACCCTGGTG 300

************************************************************

XhoIEcoRV GTGGGGAAGACTTTGGAGGAAATAACCAGTGACTTCCGAGGCTTCTACAGACTCCTGAGC 360

*enosf1b*  GTGGGGAAGACTTTAGAGGAAATAACCAGTGACTTCCGAGGCTTCTACAGACTCCTGAGC 360

************** *********************************************

XhoIEcoRV AGCGATGGTCAGATGAGATGGATCGGACCAGAGAAAGGAGTCATTCATCTGGCAACAGCA 420

*enosf1b*  AGCGATGGTCAGATGAGATGGATCGGACCAGAGAAAGGAGTCATTCATCTGGCAACAGCA 420

************************************************************

XhoIEcoRV GCCGTATTGAATGCTGTCTGGGATTTATGGGCAAGAGTAGAGAGAAAGCCCTTGTGGAAG 480

*enosf1b*  GCCGTATTGAATGCTGTCTGGGATTTATGGGCAAGAGTAGAGAGAAAGCCCTTGTGGAAG 480

************************************************************

XhoIEcoRV CTACTTGTTGACATGGATCCTGCAAAGCTGATCAGCTGTATTGATTTCAGAT-------- 532

*enosf1b*  CTACTTGTTGACATGGATCCTGCAAAGCTGATCAGCTGTATTGATTTCAGATATCTCACT 540

****************************************************

XhoIEcoRV ------------------------------------------------------------

*enosf1b*  GATGCTCTGACGGAGCAGGAAGCTTTAGATATATTGGTAAAAGGCAAAAAAGATCAGAAA 600

XhoIEcoRV ------------------------------------------------------------

*enosf1b*  AGTAGAGAGGAACAGATGTTGAAAGAAGGCTATCCTGCCTATACCACTTCCTGTGCCTGG 660

XhoIEcoRV ------------------------------------------------------------

*enosf1b*  CTGGGTTACACTGACCAGCAGCTTACACAGCTCTGCAATGAAGCTCTTGCTCAAGGATGG 720

XhoIEcoRV ------------------------------------------------------------

*enosf1b*  ACTAAATTTAAAGTGAAAGTCGGGGCTGATTTGCAGGATGATATTCGTAGATGCAGCCTT 780

XhoIEcoRV ------------------------------------------------------------

*enosf1b*  ATTCGAAAGCTGATTGGACCAAACAACACACTGATGATTGATGCCAACCAACGGTGGGAT 840

XhoIEcoRV ------------------------------------------------------------

*enosf1b*  GTTAATGAAGCAATCACCTGGGTAACCAAGCTGGCAGAGTTTCAGCCACTGTGGATTGAG 900

XhoIEcoRV ------------------------------------------------------------

*enosf1b*  GAGCCCACCTGTCCTGATGACATTCTAGGTCATGCTTCCATCTCTAAGGCACTTGCACCT 960

XhoIEcoRV ------------------------------------------------------------

*enosf1b*  TTGGGTATCGGAGTTGCCTCTGGAGAGCAGTGCCATAACAGAGTGATGTTCAAGCAGTTT 1020

XhoIEcoRV ------------------------------------------------------------

*enosf1b*  CTCCAGGCCTCAGCTCTGCAGTTTGTTCAGATTGACAGCTGTAGGGTCGGCAGTGTTAAT 1080

XhoIEcoRV ------------------------------------------------------------

*enosf1b*  GAGAACCTTGCCACTATACTCATGGCTGCCAAGTTTAATGTTCCTGTATGTCCTCATGCT 1140

XhoIEcoRV ------------------------------------------------------------

*enosf1b*  GGCGGTGTTGGACTGTGTGAGCTTGTACAGCACCTTATTTTGTTTGACTACATTTCAGTG 1200

XhoIEcoRV ------------------------------------------------------------

*enosf1b*  TCTGCTAGTTTAAGTAATAGGATGTGTGAGTTCGTGGATCACCTTCATGAACACTTCAAA 1260

XhoIEcoRV ------------------------------------------------------------

*enosf1b*  AGCCCCACCGTGATCAGAAATGCCAAATACATTCCACCAAAAGACCCTGGATTCTCTTGT 1320

XhoIEcoRV ------------------------------------------------------------

*enosf1b*  GAAATGCTGGAGGAATCTGTGAAAAAGCATCAGTATCCTGAAGGGGAGGTTTGGAGAGCC 1380

XhoIEcoRV ------------------------------------------------------------

*enosf1b*  ATTGAGAAACAGCAGAAGTGATAGAAACCAACACAAGCACTGACTGCTGAGGAAATTAAA 1440

XhoIEcoRV ------------------------------------------------------------

*enosf1b*  AATGACCAACGAAAGGGAGAAAAATACTTAAAGACCTGTCAGGTTGTCCTGATTGACTTA 1500

XhoIEcoRV ------------------------------------------------------------

*enosf1b*  AAGGGCATTATAAATGGATGGGAGAGTAAATAGGGAAATATATATTTTCTTAAACAAATG 1560

XhoIEcoRV ------------------------------------------------------------

*enosf1b*  GTTTAAACTATATGGAGTCTTTACTGTAAGTCTTTACTCTGTTTTGTCTTATTTCAATGT 1620

XhoIEcoRV ------------------------------------------------------------

*enosf1b*  TCTTAAAGGTCATACTGCACTGTGACAGGACTAGAGATCAAGTGTATGCTGACACTATTT 1680

XhoIEcoRV ------------------------------------------------------------

*enosf1b*  GTTTAATAGTCTGAATAATGACTAAAATGGGGAATTTTATCTGTGCATTAACTGAAGGGG 1740

XhoIEcoRV ------------------------------------------------------------

*enosf1b*  ACTGTATGGTATATGTACTTGGTGAATGTTACCAATCAAACTGAAAAAAACTTTTCTATT 1800

XhoIEcoRV ---------------------------------------

*enosf1b*  TTTATGCATTTCCTGACATAAATATGTAATGTTTTGGTC 1839
